# *MARCH8* suppresses hepatocellular carcinoma by promoting *SREBP1* degradation and modulating fatty acid de novo synthesis

**DOI:** 10.1038/s41419-025-07707-9

**Published:** 2025-05-16

**Authors:** Dao-yuan Tu, Rui Peng, Sheng-jie Jin, Bing-bing Su, Song-song Fan, Jia-hao Zhang, Shun-yi Wang, Yang-yang Miao, Guo-qing Jiang, Chi Zhang, Jun Cao, Dou-sheng Bai

**Affiliations:** 1 Department of Hepatobiliary Surgery, Northern Jiangsu People’s Hospital Affiliated to Yangzhou University, Yangzhou, China; 2 General Surgery Institute of Northern Jiangsu People’s Hospital, Yangzhou, China

**Keywords:** Mechanisms of disease, Tumour-suppressor proteins

## Abstract

Hepatocellular carcinoma (HCC) is one of the most prevalent malignant tumors of the digestive system, and its prevalence is currently increasing. The current study aims to elucidate the mechanism by which membrane-associated *RING-CH8* (*MARCH8*) impedes the progression of HCC. *MARCH8* was identified as a distinct prognostic marker for recurrence-free survival (RFS) and overall survival (OS) in patients with HCC. This study shows that *MARCH8* hinders lipid deposition by suppressing the expression of key enzymes for the de novo synthesis of fatty acids (FAs) via RNA sequencing, untargeted metabolomics, and a series of in vivo and in vitro experiments. Further experimental validation demonstrated that *MARCH8* was a novel E3 ligase of *sterol regulatory element binding protein 1* (*SREBP1*). And, it primarily promoted the degradation of *SREBP1*, thereby suppressing the expression of key enzymes involved in the de novo synthesis of FAs. In conclusion, this study has identified *MARCH8* as a key “switch” that can be targeted to prevent de novo FA synthesis in HCC cells. This finding may have substantial implications for discovering innovative therapeutic strategies for HCC.

## Introduction

Hepatocellular carcinoma (HCC) is the most common type of primary liver cancer, with high mortality and poor prognosis rates [1, 2]. The prevalence of HCC is strongly associated with obesity and nonalcoholic fatty liver disease [3–5]. Despite various suggested pathways such as oxidative stress, endoplasmic reticulum stress, adipokine dysregulation, increased pro-inflammatory cytokines, and disrupted gut microbiota, the precise processes underlying lipid metabolism-related hepatocarcinogenesis have yet to be fully understood [6–10].

Cancer cells have distinct metabolic changes, referred to as “metabolic reprogramming,” which promote their adaptability to the microenvironment [11]. The Warburg effect is a highly studied modification in cancer cells, but altered FA metabolism signifies another unique characteristic. Moreover, HCC has significant changes in lipid metabolism, marked by elevated lipid accumulation due to enhanced de novo FA production [12]. HCC presents significant modifications in lipid metabolism, characterized by increased lipid accumulation through increased de novo synthesis of fatty acids (FAs) [13–15]. The resulting increase in lipid accumulation within cancer tissue has been shown to protect cancer cells from oxidative damage triggered by chemotherapy [16], promote cancer cell survival, and provide energy for distant metastasis [17]. It also contributes to membrane phospholipid homeostasis [18].

Our previous studies revealed that membrane-associated *RING-CH* (*MARCH*) ligases correlated with HCC prognosis, clinicopathological characteristics, and the tumor immune microenvironment (TIM) by bioinformatics analysis [19]. Moreover, *MARCH* ligase has been shown to have an important impact on tumor formation and progression [20–22]. The protein *MARCH8* was first discovered as a cellular homologue of the viral E3 ubiquitin ligase, encoded by specific herpesviruses [23]. It has, therefore, drawn attention as an antiviral factor [24]. Recent studies have increasingly focused on the role of *MARCH8* in tumorigenesis and progression [25–27]. However, the inhibitory effect of *MARCH8* on tumor growth via its role in regulating FA metabolism has not been investigated.

The primary transcription factor involved in the regulation of lipid homeostasis [28, 29], *sterol regulatory element-binding protein 1* (*SREBP1*), a transendoplasmic reticular membrane protein, can be activated and translocated to the nucleus through specific protein hydrolysis processing. This process is triggered by changes in various FA metabolic signals, which in turn stimulate the transcription of its target genes. These genes encode key enzymes, including *stearoyl-coenzyme A desaturase1* (*SCD1*), *fatty acid synthase* (*FASN*), *acetyl-coenzyme A carboxylase 1* (*ACC1*), and *ATP citrate lyase* (*ACLY*) [30, 31]. *SREBP1* plays a pivotal role in the FA de novo synthesis in HCC cells and serves as a crucial mediator between HCC cell FA metabolism and oncogenic signaling pathways [32, 33].

This study demonstrates that *MARCH8* plays a key role in HCC. It was also found that *MARCH8* can effectively inhibit de novo FA production, thus hindering the advancement of HCC.

## Materials and methods

### Patients and sample collection

A total of 140 HCC tissues were provided by the Department of Biobank, Northern Jiangsu People’s Hospital/Northern Jiangsu People’s Hospital Affiliated to Yangzhou University between January 2015 and May 2022. The tissues were collected and processed to create tissue microarrays (TMAs), which included both tumor tissues and matched adjacent non-tumor tissues. The TMAs were obtained from Shanghai Xinchao Biotechnology Co., Ltd.

### Ethics approval and consent to participate

All methods in this study were carried out in accordance with the Declaration of Helsinki for human subjects and ARRIVE guidelines for animal research. The utilization of the aforementioned HCC samples was approved by the Ethics Committee of the Northern Jiangsu People’s Hospital Affiliated to Yangzhou University (Approval Reference Number: 2024ky338). Yangzhou University Laboratory Animal Ethics Committee (Approval Reference Number: yzu-lcyxy-n030) for studies involving live vertebrates. Written informed consent was obtained from all human participants prior to their inclusion in this study. Participants were fully informed about the research objectives, procedures, and potential risks/benefits.

### Cell lines and lentivirus infection

The human HCC cell lines HCCLM3 (termed LM3), PLC/PRF/5 (termed PLC), Hep3B, Huh7, and SK-Hep-1 were obtained from the Cell Bank of the Chinese Academy of Sciences (Shanghai, China). They report that the cell lines were authenticated using STR profiling and tested for mycoplasma contamination using PCR. All HCC cell lines were cultured in 1% streptomycin/penicillin (Beyotime, Shanghai, China) and DMEM medium (HyClone) supplemented with 10% fetal bovine serum (Gibco). The cultures were maintained at 37 degrees centigrade with 5% CO₂ in a humidified incubator. The molecules in this study lentiviral vectors, including short hairpin RNA (shRNA) lentiviral vectors and overexpression lentiviral vectors and their control vectors, were purchased from Guangzhou Ribobio Biotechnology Co., Ltd. Cell lines that demonstrated survival and stable transcription of the target gene were identified by subjecting them to a three-day puromycin (2 μg/ml, Beyotime, Shanghai, China) screening process in the culture medium.

### Immunohistochemistry

The specimens were subjected to a 4% paraformaldehyde fixative and subsequently embedded in paraffin. According to the IHC kit instructions (Absin, Shanghai, China), standard IHC staining procedures were performed. The antibodies used in this study were diluted according to the instructions (Supplementary Table 1). Depending on the antibody instructions, antigen retrieval was performed using EDTA and citrate solutions. The Histoscore (H-score) methodology was utilized by two experienced pathologists for the independent assessment of staining intensity. H-score = Σ (Pi * i), Pi: Represents the percentage of positive cells with a certain staining intensity out of all cells in the slice, quantified as 0 (no staining), 1 (less than 10%), 2 (10%–50%), 3 (50%–80%), or 4(80%–100%). i: Indicates the staining intensity, quantified as 0 (no staining), 1 (weak staining), 2 (moderate staining), or 3 (strong staining).

### The analysis of real-time quantitative PCR (qRT-PCR) and Western blotting (WB)

The WB and qRT-PCR analysis was carried out as described previously [21]. For both WB and qRT-PCR analysis, three biological replicates were performed to ensure the reproducibility and reliability of the results. The specific primers of the qRT-PCR quantitative analysis and the information of the primary and secondary antibodies in the WB experiment are shown in the Supplementary Table 1 and Supplementary Table 2. The quantification of protein expression levels from gel images was conducted using ImageJ software (version 1.8.0_345).

### Cell proliferation, migration, and invasion assay

Following a 14-day incubation period, during which 1000 cells were cultured in six-well plates, the plates were fixed with 3% paraformaldehyde solution for a period of 30 min. This was followed by a further 30 min during which the cells were stained with a 0.1% solution of crystal violet. Colony formation was assessed by photography and quantification. The CCK-8 solution (Beyotime, Shanghai, China) was carried out in accordance with the instructions, and the absorbance OD value of the enzyme-labeled cells was detected using an instrument at 570 nm. Transwell chambers in 24-well plates with or without Matrigel were used for cell migration and invasion assays. In total, 1 × 10^5^ cells were seeded in the upper chamber without serum, with 800 µL of complete medium in the bottom chamber. The fixation and staining steps are identical to those previously described.

### RNA sequencing and metabolomics data analysis

Three groups of Huh7-vector and Huh7-*MARCH8* cells were subjected to trypsin digestion and subsequent cell collection, rinsing in cold phosphate-buffered saline (PBS) on three occasions. The resulting cell pellets were then dispatched to Shanghai Meiji Biomedical Technology Co., Ltd for comprehensive RNA sequencing analysis and data interpretation.

In the same way, we sent five groups of Huh7-vector and Huh7-*MARCH8* cells to Guangzhou Kidio Biotechnology Co., Ltd for untargeted metabolomics analysis and data analysis.

### Triglyceride detection assay

The concentration of triglycerides (TG) was quantified by means of a Triglyceride Quantification Assay Kit (Beyotime, Shanghai, China), as detailed in the kit instructions. A total of 5 × 10⁶ cells were collected and subsequently combined with 1 ml of extraction reagent. The samples were then subjected to ultrasonication for a period of one minute, after which they were subjected to centrifugation at 8000 rpm at 4 degrees centigrade for a further ten minutes. The resulting supernatant was subjected to testing in strict accordance with the instructions delineated by the manufacturer.

### Cellular Nile red staining

The cells were enumerated and plated in a six-well plate at a density of 1 × 10⁵ cells per well. The cells were incubated for 15 min at room temperature with 500 nM Nile Red (MedChemExpress, NJ, USA) and 1 μg/ml 4′,6-diamidino-2-phenylindole (DAPI) (Beyotime, Shanghai, China). The images were captured with a fluorescence microscope and subsequently analyzed using the ImageJ software, version 1.8.0_345. In order to conduct a flow cytometry analysis, the cells were stained with Nile Red for a period of 15 min and the fluorescence intensity of each group was subsequently measured.

### Nucleus–cytoplasmic fractionation assay

Strictly follow the manufacturer’s operating (Beyotime, Shanghai, China) instructions for the experiment, in short, adherent cells were scraped, and the cell pellet was obtained through centrifugation. Subsequently, the cell pellet should be resuspended in Pre-Extraction Buffer and transferred to a microcentrifuge vial. Following vortexing and centrifugation, the cytoplasmic extract should be carefully removed from the nuclear pellet. The nuclear pellet should then be treated with an extraction buffer containing DTT and PIC. It is possible to further sonicate the extract in order to enhance the extraction of nuclear proteins. The suspension should then be subjected to centrifugation for a period of 10 min at a speed of 14,000 × *g* at 4 degrees centigrade, after which the supernatant should be transferred to a new microcentrifuge vial.

### *SREBP1* DNA binding activity assay

In order to ascertain the intracellular *SREBP1* DNA binding activity, the *SREBP1* Transcription Factor Assay Kit (Abcam, UK) was employed. The nuclear extracts were prepared using a Nuclear Extraction Kit (Abcam, UK) in accordance with the manufacturer’s instructions. The resulting nuclear proteins were then added to the wells that had been pre-coated with a specific double-stranded DNA sequence encompassing the peroxisome proliferator response element. Subsequently, the specific primary antibodies and horseradish peroxidase-conjugated secondary antibodies were added in accordance with the instructions provided. Subsequently, following the addition of the developing and halting solutions, the absorbance at 450 nm was quantified using a microplate reader.

### Coimmunoprecipitation (co-IP) and mass spectrometry

A lysis buffer containing 1 mM PMSF is added to 10⁷ cells, resulting in a lysate that is then sonicated for 5 s and centrifuged at 14,000 × *g* for 10 min. The supernatant obtained is mixed with 5 μg of primary antibody and incubated overnight. Protein samples obtained according to the standard procedure of the kit of co-IP (Absin, Shanghai, China) were subjected to WB and sent to Shanghai Jikai Biotechnology Co., Ltd. for mass spectrometry analysis. The SRE motif from the *ACC1* and *FASN* promoters was predicted using the online tool available at https://epd.epfl.ch/index.php.

### Immunofluorescence

Cells were deposited onto microscope slides and subsequently fixed in a solution of 4% paraformaldehyde. To enhance accessibility for antibodies, the cells underwent permeabilization treatment with 0.1% Triton X-100 for 10 min. Following this step, non-specific binding sites were blocked with 10% goat serum. After overnight incubation at 4 degrees centigrade with primary antibodies, the cells were rinsed with PBS. Subsequently, the cells were stained with secondary antibodies, goat anti-rabbit labeled with Alexa Fluor 555 and goat anti-mouse labeled with Alexa Fluor 488 for an hour at ambient temperature. To complete the staining protocol, the cells were counterstained with DAPI. Finally, high-definition images of the stained cells were captured scanning confocal microscope.

### Mouse xenograft model

Nude mice (4 weeks old, 15–21 g) were obtained from the Translational Medical Center at Yangzhou University. To create a subcutaneous tumor model (six nude mice per group), a suspension comprising 5 × 10⁶ cells was introduced into the dorsal region of nude mice via injection. Subcutaneous tumor volumes were measured every 3 days using calipers. Nude mice were randomly assigned using a computer-generated randomization sequence. This process was performed prior to the start of the experiment to minimize bias. Mice were sacrificed after 3 weeks, and tumor weights were measured. To detect the formation of lipid droplets, the tissues of a mouse subcutaneous tumor were collected for frozen sections and incubated in Oil Red O solution (Solarbio, Beijing, China). For the establishment of an orthotopic tumor model, mice were first anesthetized with isoflurane to ensure a pain-free procedure. Subsequently, the liver was exposed, and the cell suspension (5 × 10^6^ cells) was injected directly into the liver parenchyma. Following the injection, the surgical site was sutured and thoroughly disinfected to prevent infection. Fluorescent images of tumors were taken using an in vivo imaging system. The model of lung metastasis was generated by injecting cells (2 × 10^6^ cells) into mice via the tail vein. After 21 days, the mice were sacrificed, and the lungs were harvested, photographed, and stained with haematoxylin-eosin (H&E). Fatostatin (30 mg/kg; 150 μl; MedChemExpress, NJ, USA) was injected intraperitoneally into mice every other day based on the dose recommended in the instructions. The research project has been granted approval by the Animal Utilization Committee of Yangzhou University.

### Bimolecular fluorescence complementation (BiFC) experiment

To investigate the interaction between *MARCH8* and *SREBP1* in the HCC cells, we employed the BiFC technique. In this method, *MARCH8* was fused with the VC155 fragment, and *SREBP1* was fused with the VN173 fragment of a fluorescent protein. The corresponding plasmids were constructed using standard cloning techniques. These plasmids were then transfected into Huh7 and PLC cells using Lipofectamine 2000 reagent, following the manufacturer’s protocol. After 24 h of incubation, cells were analyzed under a fluorescence microscope to detect any restored fluorescence, which only occurs when *MARCH8* and *SREBP1* interact. Control groups, including an empty vector and single plasmid transfections (*MARCH8*-VC155 and *SREBP1*-VN173), were used to ensure the specificity of the fluorescence signals. Additionally, confocal microscopy was utilized to further examine the subcellular localization of the interaction.

### Construction of domain mutants

Firstly, we retrieved and obtained the complete amino acid sequences of *MARCH8* and *SREBP1* from the UniProt database (https://www.uniprot.org/). Next, we used the SMART database (Simple Modular Architecture Research Tool, http://smart.embl-heidelberg.de/) to predict the protein domains within the sequences of *MARCH8* and *SREBP1*. Based on the prediction results from SMART, we identified the domains of interest within *MARCH8* and *SREBP1* and designed corresponding domain-deleted mutants. The designed sequences of domain-deleted mutants were submitted to Guangzhou Ribobio Biotechnology Co., Ltd, and the plasmid was constructed.

### Glutathione S-transferase pull-down assay

The GST-tagged *SREBP1* (GST-*SREBP1*) and 3*flag-tagged *MARCH8* (flag-*MARCH8*) proteins were expressed in BL21 (DE3) Escherichia coli via the transformation of the pGEX-4T-1-GST-*SREBP1* and pET24a-3*flag-*MARCH8* plasmids, respectively. Subsequently, the E. coli was harvested, disrupted by sonication, and purified using complete flag-Tag Purification Resin (Roche) to obtain the purified flag-*MARCH8* protein. The expression and immobilisation of the GST-*SREBP1* protein were carried out using BeyoGold™ GST-tag Purification Resin, in accordance with the instructions provided by the manufacturer (Beyotime, Shanghai, China). The *SREBP1*-beads complexes were washed with GST pull-down binding buffer (10 mM MyCl2, 1 mM EDTA, 200 mM NaCl, 50 mM Tris-HCl, 1% NP-40, 1 mM DTT, pH 8.0) and incubated with purified flag-*MARCH8* at 4 degrees Celsius for 4 h on a rotating platform. Subsequently, the beads were washed and analyzed by Western Blot. The GST-tagged *MARCH8* (GST-*MARCH8*) and 3*HA-tagged *SREBP1* (HA-*SREBP1*) proteins were verified by the same method.

### Chromatin immunoprecipitation (ChIP) assays

A total of 1 × 10^7^ cells should be cross-linked with 1% formaldehyde for a period of 10 min, after which time the reaction should be quenched with 0.2 g glycine. Chromatin immunoprecipitation (ChIP) was performed using the Chromatin IP kit (Cell Signaling Technology, USA). The qRT-PCR was employed to determine the extent to which *SREBP1* binding was enriched within the promoter region. The specific primers of the ChIP quantitative analysis are shown in the Supplementary Table 3

### Statistics and reproducibility

To ascertain the mean values of multiple groups, a one-way analysis of variance with post-hoc pairwise comparison analysis was employed. The Student’s *t*-test (unpaired, two-tailed) and the Wilcoxon-test (paired two-samples) were employed for the purpose of comparing the mean value of two groups. In order to evaluate the relationship between *MARCH8* and *ACC1*, *FASN,* and *SREBP1* mRNA expression levels in human HCC tissues, the Pearson correlation coefficient was used as the statistical tool of choice. The plotted bars and error bars represent the mean, with the standard deviation indicated in parentheses, derived from replicate measurements. A *p*-value of less than 0.05 is deemed to be statistically significant. For statistical evaluation, the data were processed with SPSS version 21.0 (SPSS, Inc., Chicago, IL, USA).

## Results

### Downregulation of *MARCH8* associates with poor prognosis in HCC patients

**Fig. 1:**
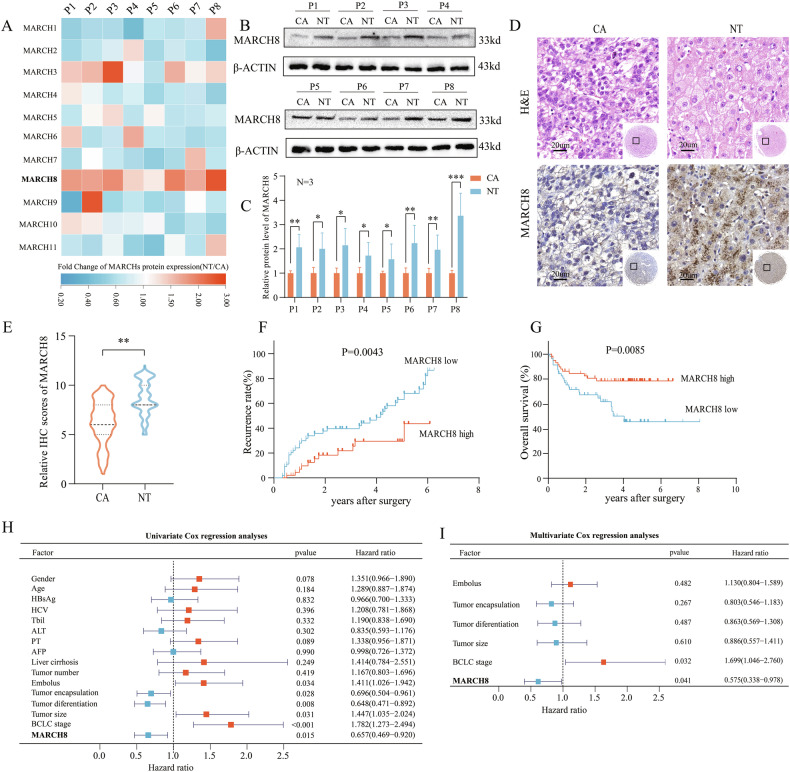
*MARCH8* is significantly downregulated in HCC cancer tissues and is associated with theprognosis of HCC patients. **A** The heatmap analysis of the folding changes (CA/NT) in *MARCH* family protein expression in the HCC cancer tissues and matched non-tumor tissues. CA: HCC cancer tissues, NT: non-tumor tissues. WB quantification was performed using ImageJ. **B**, **C** WB was performed on 8 pairs of matched NT and CA tissues. **D** TMAs were used to analyze and score *MARCH8* expression in 140 patients with HCC. **E** H-score revealed the expression level of *MARCH8* protein in HCC cancer tissues and matched non-tumor tissues. **F**, **G** Kaplan–Meier analysis examined the OS and RFS in HCC patients with high and low expression of *MARCH8*. **H**, **I** Univariate and multivariate Cox regression analyses evaluated the independent prognostic risk of OS in HCC patients. *: *p* < 0.05, **: *p* < 0.01, ***: *p* < 0.001.

Considering that the *MARCH* family comprises E3 ubiquitin ligases, which function at the protein level, the expression of the *MARCH* family was examined in the malignant and paracancerous tissues of 8 HCC patients via western blot (WB) analysis. We found that the difference between *MARCH8* was most pronounced in HCC carcinoma and non-tumor tissues (Fig. 1A–C; Supplementary Fig. 1A, B). Next, *MARCH8* was further investigated by immunohistochemical (IHC) staining, which was performed on 140 pairs of HCC cancer tissues and matched non-tumor tissues using tissue microarrays (TMAs) (Fig. 1D). The findings revealed a significant reduction in the protein expression level of *MARCH8* in HCC tumor tissues compared to adjacent tissues (Fig. 1E). Then HCC patients were divided into *MARCH8* low expression (*n* = 72) and high expression (*n* = 68) groups based on the IHC scores of HCC tumor tissues (Supplementary Fig. 1C). Kaplan–Meier survival analysis demonstrated that patients exhibiting high expression levels of *MARCH8* had significantly prolonged RFS and OS compared to those with low expression levels (Fig. 1F, G). The next step was analyzing the association between *MARCH8* expression levels and the clinical pathological characteristics identified in HCC patients. The univariate Cox proportional hazards regression analysis identified *MARCH8* (*p* = 0.015), embolus (*p* = 0.034), tumor encapsulation (*p* = 0.028), tumor differentiation degree (*p* = 0.008), tumor size (*p* = 0.031), and Barcelona Clinic Liver Cancer (BCLC) stage (*p* < 0.001) as predictive variables (Fig. 1H). However, multivariate Cox regression analysis demonstrated that the BCLC stage and the expression of *MARCH8* were identified as independent prognostic factors for OS (Fig. 1I). The findings indicate that *MARCH8* may be a significant contributor to HCC. Similarly, further investigation is required to determine whether it influences the progression of HCC.

### *MARCH8* suppresses invasive and metastatic behaviors of HCC cells in vitro and in vivo

**Fig. 2:**
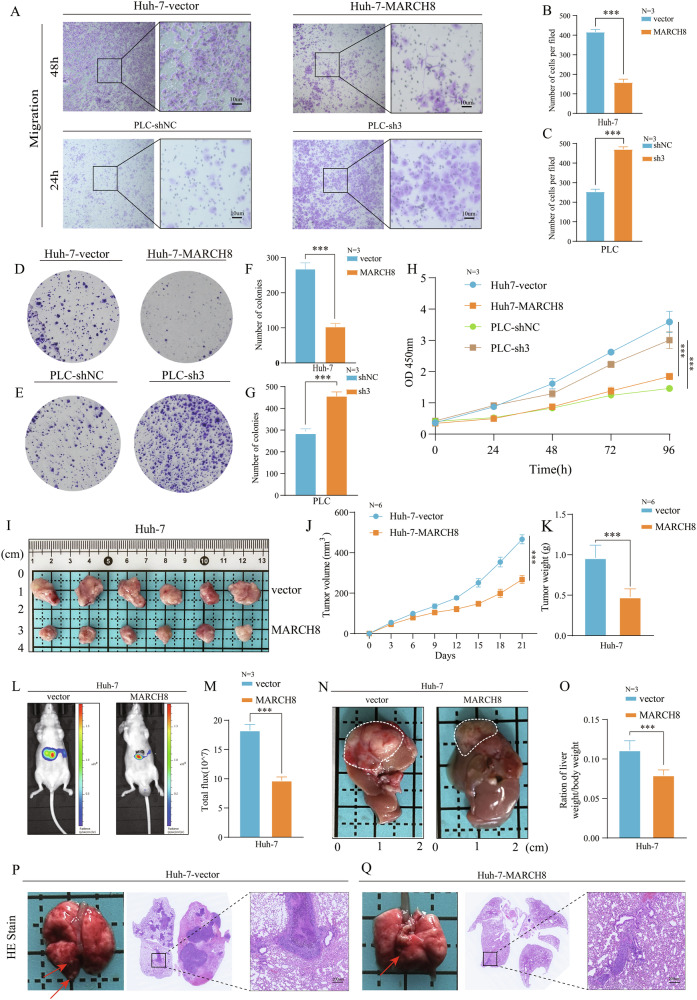
*MARCH8* can markedly suppress the invasion and metastasis of HCC cells. **A**–**C** Effect of *MARCH8* on cell migration via transwell assays. Cell proliferation was detected by Colony formation (**D**–**G**) and CCK-8 assays (**H**). Huh7-vector or Huh7-*MARCH8* cells were injected into the dorsal subcutis or liver parenchyma of nude mice. Subcutaneous tumor volumes were measured every 3 days. Tumor images (**I**), growth curves (**J**), and weights (**K**) were obtained on day 21 after dissection. **L**, **M** A live imaging system assessed tumor growth in vivo by measuring fluorescence signals. **N**, **O** The ratio of orthotopic tumor to body weight of mice in the orthotopic model. **P**, **Q** Huh7-vector and Huh7-*MARCH8* cells were injected into mice *via* the tail vein. After 21 days, all mice were euthanized, and their lungs were dissected and collected. These lung sections were stained with H&E and scanned. ***: *p* < 0.001.

The expression of *MARCH8* was analyzed in 5 HCC cell lines: LM3, PLC, Huh7, SK-Hep1, and HEP3B. The findings indicated that *MARCH8*’s expression levels were lower in Huh7 cells and higher in PLC cells (Supplementary Fig. 2A–C). Next, the molecular function of *MARCH8* was determined in HCC. The *MARCH8* cDNA vector lentivirus was used to transfect Huh7 cells (Supplementary Fig. 2D–F), whereas the *MARCH8*-shRNA lentivirus (sh1, sh2, and sh3) was transfected into PLC cells. However, sh3 was designated for further experiments (Supplementary Fig. 2G–I). Matrigel invasion experiments revealed that *MARCH8* knockdown enhanced the invasion and migration of PLC cells (Supplementary Fig. 2J–L), whereas its overexpression significantly suppressed invasion and migration in Huh7 cells (Fig. 2A–C). The colony formation (Fig. 2D–G) and CCK-8 (Cell Counting Kit-8) (Fig. 2H) experiments demonstrated that *MARCH8* overexpression markedly diminished the proliferative capacity of HCC cells, whereas its knockdown markedly enhanced their proliferation. To investigate the function of *MARCH8* in HCC cells in vivo, *MARCH8*-overexpressing Huh7 cells and *MARCH8* knockdown (sh3) PLC cells were subcutaneously transplanted into nude mice. The mice harboring *MARCH8*-overexpressing Huh7 cells had reduced tumor sizes and weights relative to those harboring control vector-infected cells (Fig. 2I–K), and mice harboring sh3 PLC cells exhibited increased tumor sizes and weights compared to those harboring cells with control vector-infected cells (Supplementary Fig. 2M–O). An orthotopic tumor model was developed in nude mice to better validate these findings. The results demonstrated that the fluorescence signal of tumors in mice overexpressing *MARCH8* was markedly reduced compared to the negative control group (Fig. 2L, M). Furthermore, the liver-to-body weight ratio was significantly reduced in the *MARCH8* overexpression group (Fig. 2N, O). Moreover, the study evaluated lung metastasis using H&E staining. The results demonstrated that *MARCH8* overexpression impeded lung metastasis of HCC cells in vivo (Fig. 2P, Q). Overall, evidence indicates that *MARCH8* plays a suppressive role in tumorigenesis, where its overexpression is associated with reduced tumor burden, whereas its depletion leads to more aggressive tumor growth. These findings highlight the potential of *MARCH8* as a therapeutic target in modulating cancer progression.

### Role of *MARCH8* in suppressing lipid accumulation in HCC

**Fig. 3:**
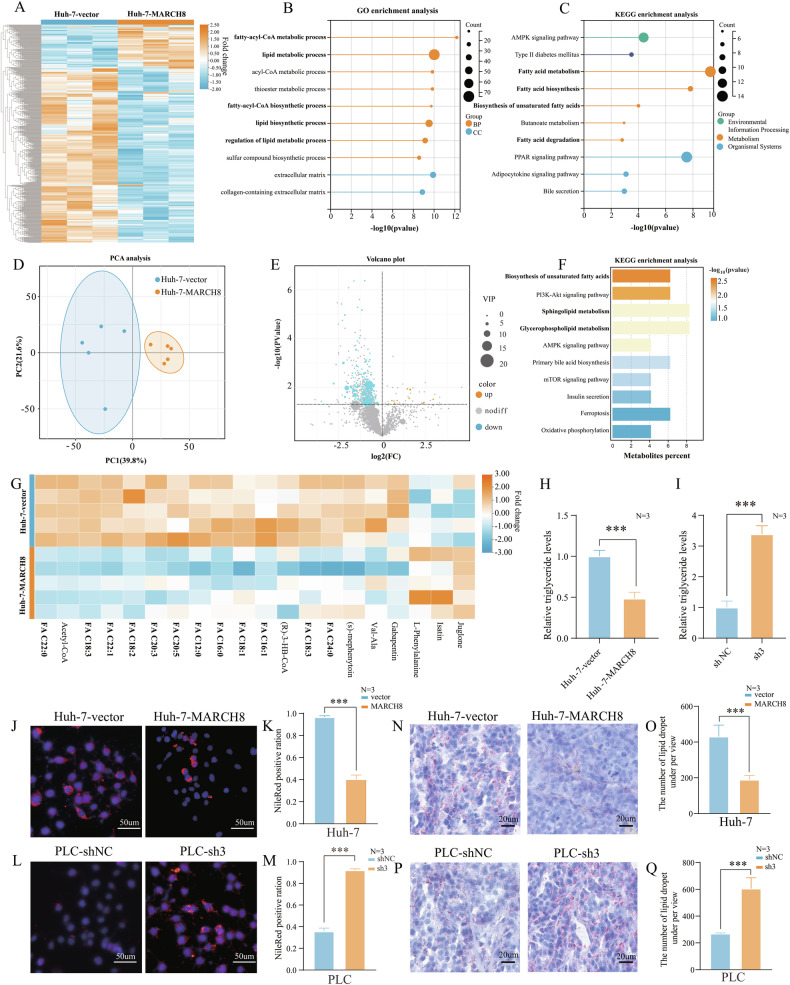
*MARCH8* is closely related to lipid metabolism in HCC cells. **A** RNA sequencing of Huh7-vector and Huh7-*MARCH8* groups identifies 586 differentially expressed genes (DEGs). **B**, **C** GO, and KEGG enrichment analyses reveal *MARCH8*’s close association with lipid metabolism based on DEGs. **D** Principal Component Analysis (PCA) of non-targeted metabolomics in Huh7-vector and Huh7-*MARCH8* samples. **E** The volcano plot shows significant downregulation of metabolites in the *MARCH8* overexpression group compared to the control group. **F** KEGG enrichment analysis for differential metabolites. **G** Heatmap of top 20 metabolites with the most significant differences. **H** Relative TG levels in Huh7-vector and Huh7-*MARCH8* cells. **I** Relative TG levels in PLC-shNC and PLC-sh3 cells. **J**, **K** Nile Red and DAPI staining show lipid droplets (red) and nuclei (blue) in Huh7-vector or Huh7-*MARCH8* cells. **L**, **M** Nile Red and DAPI staining show lipid droplets (red) and nuclei (blue) in PLC-shNC and PLC-sh3 cells. **N**–**Q** Oil Red O staining highlights lipid droplets in tumors. ***: *p* < 0.001.

To further investigate the potential biological role of *MARCH8* in HCC, RNA sequencing was performed on *MARCH8* overexpression and control vector Huh7 cells. It was found that approximately 586 genes were significantly changed (Fig. 3A). Among the top 30 differentially expressed genes (DEGs), several lipid metabolism-related genes emerged as responsive genes sensitive to *MARCH8* overexpression (Supplementary Fig. 2P). Based on these DEGs, the enrichment of biological processes in the Gene Ontology (GO) (Fig. 3B) and the enrichment in the Kyoto Encyclopedia of Genomes (KEGG) (Fig. 3C) were presented in graphical form. The enrichment pathway contains multiple lipid metabolism pathways, including lipid metabolism, lipid biosynthesis, FA metabolism, FA biosynthesis, and FA degradation. Therefore, this study showed that the overexpression of *MARCH8* may be associated with lipid metabolism *via* GO and KEGG enrichment analyses. To further verify the biological phenomenon of significant changes in lipid metabolism by *MARCH8*, liquid chromatography-mass spectrometry (LC–MS) untargeted metabolomics analysis was performed in *MARCH8* overexpression and control vector Huh7 cells (Fig. 3D). A total of 534 differential metabolites were identified, and interestingly, most of them were significantly downregulated in the *MARCH8* overexpression group (Fig. 3E). It was evident that 22.01% of the identified metabolites were lipids. The KEGG enrichment analysis was then conducted based on differential metabolites, and it was found that the metabolites were enriched in lipid metabolic pathways, including the biosynthesis of unsaturated FAs, sphingolipid metabolism, and glycerophospholipid metabolism (Fig. 3F). More importantly, untargeted lipidomic analysis demonstrated that overexpression of *MARCH8* in HCC cells led to a significant reduction in FA levels. Notably, the top 20 differential metabolites included oleic acid (C18:1), palmitic acid (C16:0), lauric acid (C12:0), palmitoleic acid (C16:1), and eicosapentaenoic acid (C20:5) (Fig. 3G). These data suggest that *MARCH8* may promote lipid accumulation by negatively regulating FA metabolism. Next, this study also investigated the triglyceride (TG) content of HCC cells and discovered that *MARCH8* overexpression significantly reduced the accumulation of TG in Huh7 cells (Fig. 3H), whereas suppression of *MARCH8* considerably raised the level of TG in PLC cells (Fig. 3I). To validate these results, fluorescence microscopy showed that Nile red staining intensity was reduced in *MARCH8* overexpression cells and increased in *MARCH8* knockdown cells (Fig. 3J–M). Similar to Nile red staining, Oil Red O staining of mouse tumor tissues showed a significant decrease in the number of lipid droplets after *MARCH8* overexpression and, however, a substantial increase in the number of lipid droplets after its knockdown (Fig. 3N–Q). Collectively, these results provide compelling evidence that *MARCH8* may play a role in promoting lipid accumulation in HCC by negatively regulating FA metabolism.

### *MARCH8* reshaping FA de novo synthesis by inhibiting ACC1 and FASN

**Fig. 4:**
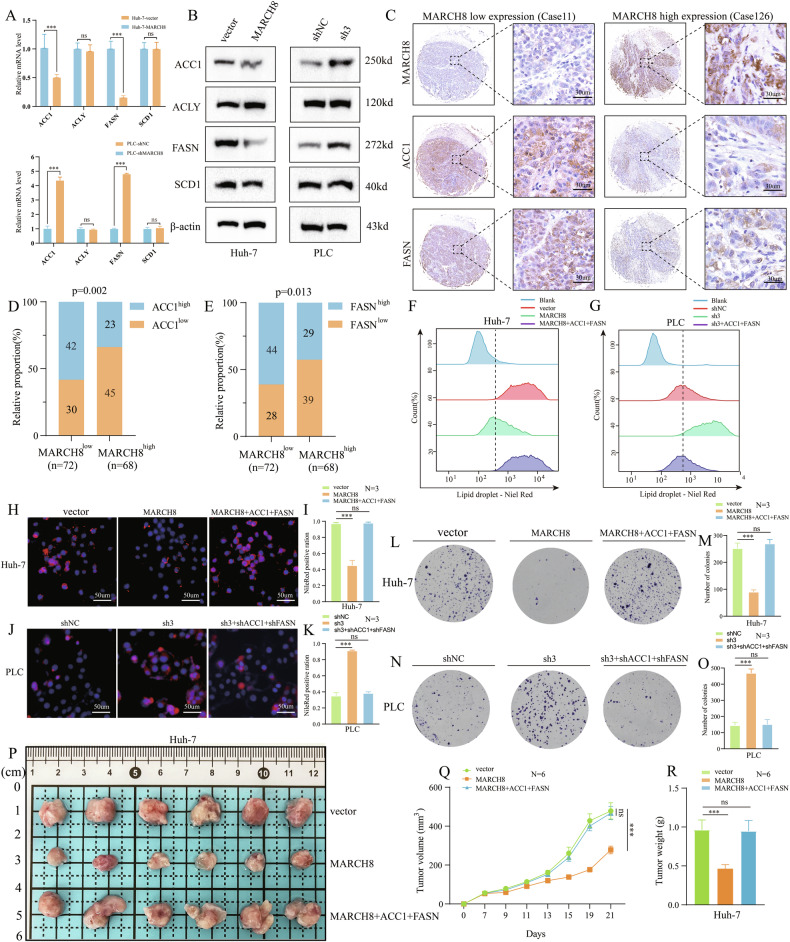
*MARCH8* inhibits lipid accumulation and HCC progression by downregulating key enzymes for FA synthesis. **A** qRT-PCR and **B** Western blot analyses of *ACC1*, *ACLY*, *FASN*, and *SCD1* in Huh7-vector and Huh7-*MARCH8*. **C** Representative IHC staining of *MARCH8*, *ACC1*, and *FASN* in HCC TMAs. **D**, **E** Correlation of *MARCH8* with *ACC1* and *FASN* protein and mRNA expression in HCC TMAs based on H-score. **F**, **G** The fluorescence intensity of the Nile Red staining was determined by flow cytometry. **H**, **I** Nile Red and DAPI staining show lipid droplets and nuclei in Huh7-vector and Huh7-*MARCH8* cells. **J**, **K** Nile Red, and DAPI staining show lipid droplets and nuclei in PLC-shNC and PLC-sh3 cells. **L**–**O** Colony formation assay for cell proliferation. **P** Image of dissected subcutaneous tumors from different groups. **Q** Nude mouse tumor growth curve and **R** weight analysis. ns: non-significant, ***: *p* < 0.001.

FA metabolism exerts a pivotal influence on HCC progression through three principal processes: FA synthesis, FA oxidation, and FA uptake and transport [34]. To better understand the regulatory function of *MARCH8* in FA metabolism, the current study investigated its effect on the expressions of key enzymes associated with FA synthesis (*ACC1*, *ACLY*, *FASN*, and *SCD1*), FA β-oxidation (*ACOX1*, *ACOX2*, *CPT1A*, and *CPT1B*), and FA uptake catabolism (CD36). In the results, *MARCH8* overexpression significantly reduced *FASN* and *ACC1*’s relative mRNA and protein expressions, whereas its suppression significantly increased them (Fig. 4A, B; Supplementary Fig. 3A, B). However, the suppression and overexpression of *MARCH8* did not affect the expression of the key enzymes involved in FA β-oxidation and FA absorption (Supplementary Fig. 3C–G). Further, IHC labeling of subcutaneous xenograft tumor tissue demonstrated that *MARCH8* overexpression suppressed the expression of *ACC1* and *FASN* (Supplementary Fig. 3H). To further explain the possible relationship between *ACC1* and *FASN* expression and *MARCH8* expression in HCC tissues, TMAs were used for IHC labeling (Fig. 4C). The results showed a negative association between *MARCH8* protein expression and that of *ACC1* (*p* = 0.002) and *FASN* (*p* = 0.013) (Fig. 4D, E). The aforementioned results indicate that *MARCH8* may have prevented the FA synthesis in HCC cells by suppressing the expression of *FASN* and *ACC1*.

The study further investigated whether *MARCH8* inhibits FA synthesis and HCC cell progression by suppressing the expression of *ACC1* and *FASN*. To establish stable cell lines, *ACC1* and *FASN* lentiviruses were transfected into Huh7 cell lines that overexpress - *MARCH8* expression (Supplementary Fig. 3I, K, M). Similarly, *ACC1* and *FASN* lentiviruses were knocked down into PLC cell lines that knockdown *MARCH8* expression (Supplementary Fig. 3J, L, N). In Huh7 cell lines, the reduction in lipid droplet content due to *MARCH8* overexpression was reversed by the co-overexpression of *ACC1* and *FASN*, as shown by flow cytometry and fluorescence microscopy (Fig. 4F, H, I). In PLC cell lines, the increase in lipid droplet content observed upon *MARCH8* knockdown was similarly reversed by the co-knockdown of *ACC1* and *FASN*, as demonstrated by flow cytometry and fluorescence microscopy (Fig. 4G, J, K). Next, a colony formation experiment was performed to show that simultaneous co-overexpression of *ACC1* and *FASN* can restore the decreased proliferation ability of cells caused by *MARCH8* overexpression (Fig. 4L, M), while simultaneous co-knockdown of *ACC1* and *FASN* can reverse the increased proliferation ability after *MARCH8* knockdown (Fig. 4N, O). To determine whether *MARCH8* exerts its tumor-suppressive effects by modulating the expression of *ACC1* and *FASN*, xenograft tumor experiments were performed using the abovementioned cell lines (Fig. 4P). As anticipated, the simultaneous overexpression of *ACC1* and *FASN* in Huh7 cells overexpressing *MARCH8* can reverse the inhibitory effect of *MARCH8* overexpression on tumor formation (Fig. 4Q, R).

### *MARCH8*-mediated regulation of *SREBP1* in FA metabolism in HCC

**Fig. 5:**
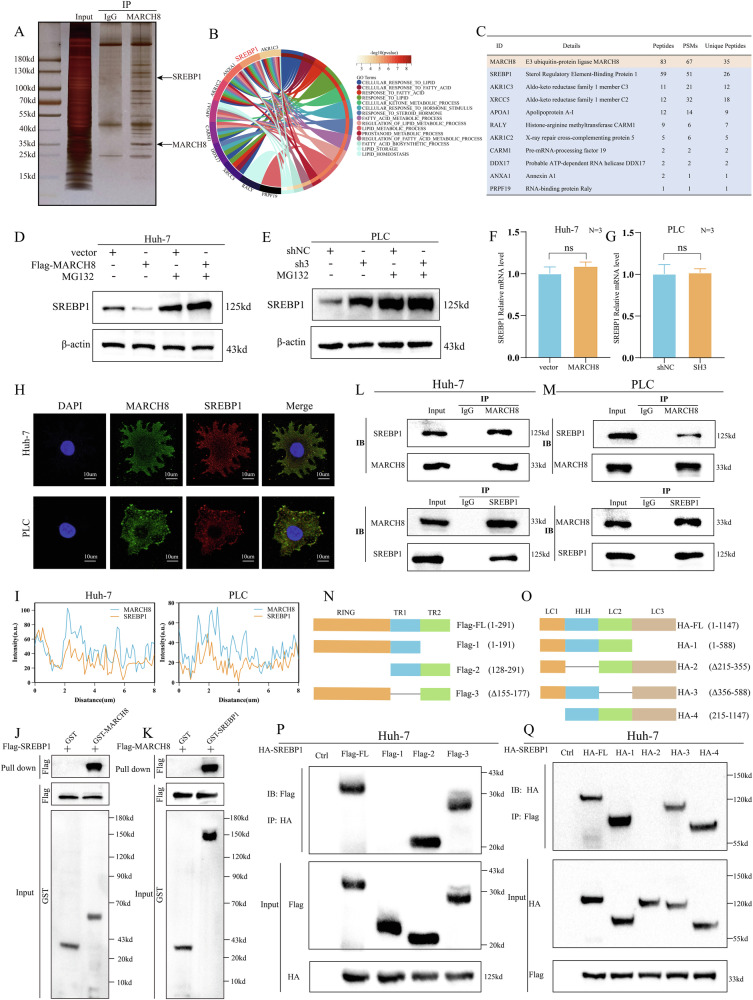
*MARCH8* specifically interacted with *SREBP1* protein. **A** Silver staining confirms the *MARCH8* complex after IP assay. **B** GO enrichment analysis based on proteins in mass spectra and listing of pathways associated with lipid metabolism. **C** Top 10 potential interacting proteins associated with lipid metabolism identified by mass spectrometry. WB analysis of *SREBP1* levels in **D** Huh7-*MARCH8* and **E** PLC-sh*3* cells treated with MG132 (10 µM for 6 h). **F**, **G** qRT-PCR assessment of *SREBP1* mRNA expression after *MARCH8* overexpression or knockdown. **H**, **I** Immunofluorescence shows co-localization of *MARCH8* and *SREBP1* in Huh7 and PLC cells, with fluorescence intensity profiles plotted using ImageJ. **J**, **K** Interaction between *MARCH8* and *SREBP1* was determined by GST precipitation, and purified GST was used as a control. **L**, **M** Co-IP assay in Huh7 and PLC cells confirms *MARCH8-SREBP1* interaction. **N**, **O** Structural domains of *MARCH8* and *SREBP1* deletion mutants were analyzed for binding specificity using UniProt and SMART databases. **P**, **Q** Co-IP assays clarify specific binding domains of *MARCH8* and *SREBP1* in Huh7 cells. ns: non-significant.

*MARCH8* functions as an E3 ubiquitin ligase. Co-IP experiments were conducted to determine whether it can directly bind to *FASN* and *ACC1* to promote their ubiquitination and degradation. However, the results contradicted this hypothesis (Supplementary Fig. 4A, B). To investigate the proteins that *MARCH8* can specifically bind to directly, immunoprecipitation (IP) was conducted, followed by silver staining, as per the standard method (Fig. 5A). A total of 72 proteins were detected through gel band mass spectrometry after excluding proteins from the IgG group. After this, GO enrichment analyses further identified the top 10 proteins associated with lipid metabolism (Fig. 5B, C). Next, the study evaluated whether *MARCH8* regulates the protein levels of these 10 proteins through ubiquitin-mediated degradation pathways. Notably, the results demonstrated that changes in *SREBP1* protein levels were exclusively observed in *MARCH8*-overexpressing Huh7 cells and *MARCH8* knockdown PLC cells. These alterations were reversible following MG132 treatment (Fig. 5D, E; Supplementary Fig. 4C, D). Additionally, no significant changes were detected in other proteins (Supplementary Fig. 4E–H). Moreover, *MARCH8* did not affect the mRNA levels of *SREBP1* (Fig. 5F, G), suggesting that *MARCH8* may have a potential regulatory role in post-translational modifications of *SREBP1*. Thus, *SREBP1* was regarded as a possible target of *MARCH8*. Both can co-localize effectively in the cytosol of Huh7 and PLC cells, as demonstrated by immunofluorescence experiments (Fig. 5H, I). By utilizing the BiFC technique, we selected the YN155 and VC173 fragments of the Venus fluorescent protein and fused them with the target proteins *MARCH8* and *SREBP1* for expression, respectively. Through observation under a fluorescence microscope, fluorescent signals were detected in the transfected Huh7 and PLC cells (Supplementary Fig. 4I, J). The next step involved the construction of the proteins GST-*MARCH8* and GST-*SREBP1* for in vitro pull-down experiments with purified recombinant proteins. As predicted, *MARCH8* is directly bound to *SREBP1* (Fig. 5J, K). Further, the co-IP assay verified that *MARCH8* and *SREBP1* can bind specifically (Fig. 5L, M). To further explore the specific regions where these two proteins, *MARCH8* and *SREBP1*, interact with each other, a truncated mutant of *MARCH8* was established as per the UniProt and SMART databases (Fig. 5N, O). These co-IP assays indicated that the truncated mutants of *MARCH8*, which lacked the TR2 domains, showed less interactions with *SREBP1* in Huh7(Fig. 5P) and PLC (Supplementary Fig. 4K) cells. Similarly, it was found that *SREBP1* truncation mutants that lack the LC2 structural domain demonstrated reduced interactions with *MARCH8* in Huh7(Fig. 5Q) and PLC (Supplementary Fig. 4L) cells. This evidence suggests that the TR2 and LC2 domains are the dominant components in the interaction between *MARCH8* and *SREBP1*.

### *MARCH8* promotes the degradation of the *SREBP1* protein via ubiquitination

**Fig. 6:**
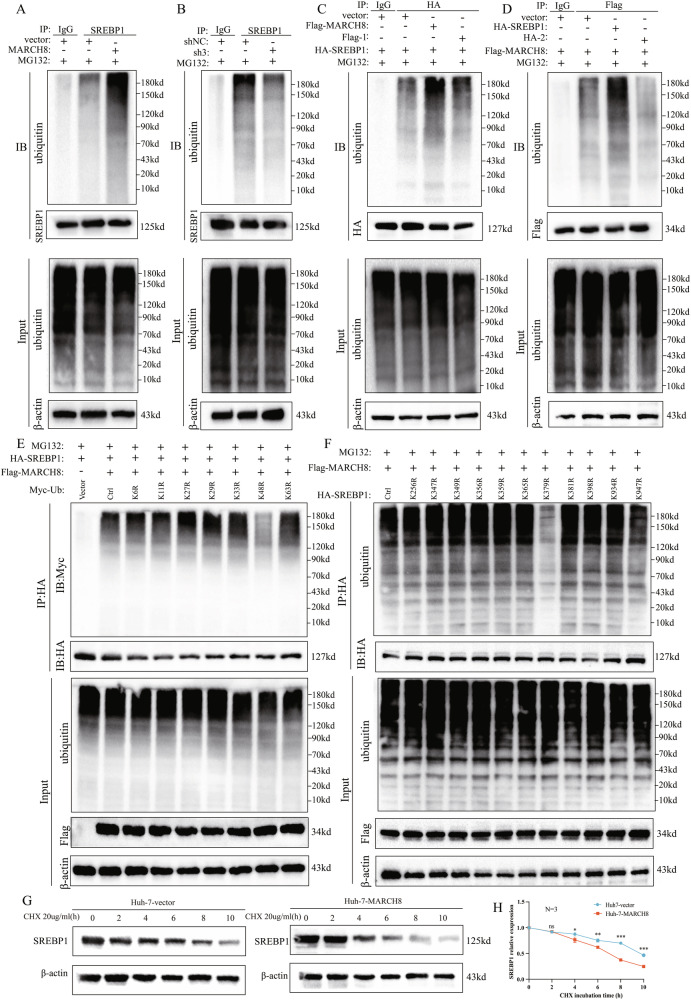
*MARCH8* enhances *SREBP1* ubiquitination levels. Ubiquitination assays of *SREBP1* in **A** Huh7-*MARCH8* and **B** PLC-sh3 cells. **C**, **D** Ubiquitination assays in HEK293T cells transfected with Flag-*MARCH8* (**C**) or HA-*SREBP1* (**D**). **E** Co-transfection of 7 mutant ubiquitin Myc plasmids with Flag-*MARCH8* and HA-*SREBP1* for 48 h in HEK293T cells, followed by co-IP assays with anti-HA microbeads. **F** Co-transfection of HA-*SREBP1* plasmids with mutated lysine sites and Flag-*MARCH8* in HEK293T cells for 48 h, followed by co-IP assays. All ubiquitination experiments were treated with MG132 (10 µM for 6 h). **G**, **H** WB analysis shows reduced *SREBP1* protein levels at specified time points after cycloheximide (20 µg/mL) treatment in Huh7 cells. ns: non-significant, *: *p* < 0.05, **: *p* < 0.01, ***: *p* < 0.001.

The present study conducted ubiquitination experiments to investigate whether *MRARC8* regulates the degradation of *SREBP1*. The polyubiquitination level of *SREBP1* was significantly elevated in Huh7 cells of *MRARC8* overexpressing (Fig. 6A). However, the knockdown of *MRARC8* reduced the ubiquitination of *SREBP1* in PLC cells (Fig. 6B). Furthermore, the ubiquitination level of *SREBP1* was found to be reduced as a result of mutations in the TR2 domain of *MRARC8* or the LC2 domain of *SREBP1*, which further demonstrates the crucial function of the two structural domains in regulating the degradation of *SREBP1* by *MRARC8* (Fig. 6C, D). To evaluate the precise type of ubiquitination catalyzed by *MRARC8*, a series of ubiquitin lysine mutants (K6, K11, K27, K29, K33, K48, and K63) were constructed and analyzed *via* IP assays (Fig. 6E; Supplementary Fig. 5A). The findings indicate that *MARCH8* can ubiquitinate and degrade *SREBP1 via* proteasome-mediated degradation initiated by K48-linked ubiquitination. Next, this study developed a series of lysine mutants that were constructed from the potential lysine sites of *SREBP1* [35] to determine which lysine sites are implicated in the *MARCH8* ubiquitination process. Subsequently, IP assays were conducted, and Lys-379 was found to be a potential key site for the *MARCH8* ubiquitination of *SREBP1* (Fig. 6F). Furthermore, the effect of cycloheximide (CHX), an inhibitor of protein translation, was explored on *SREBP1* protein levels. Its treatment resulted in a lowered half-life of the *SREBP1* protein in Huh7 cells of *MARCH8*-overexpressing (Fig. 6G, H), whereas the knockdown of *MARCH8* led to the stabilization of the *SREBP1* protein in PLC cells (Supplementary Fig. 5B, C). The evidence indicates that *MARCH8* engages in direct interaction with *SREBP1*, functioning as an *SREBP1* E3 ubiquitin ligase within HCC cells.

### *MARCH8* inhibits ACC1 and FASN expression by degrading *SREBP1*

**Fig. 7:**
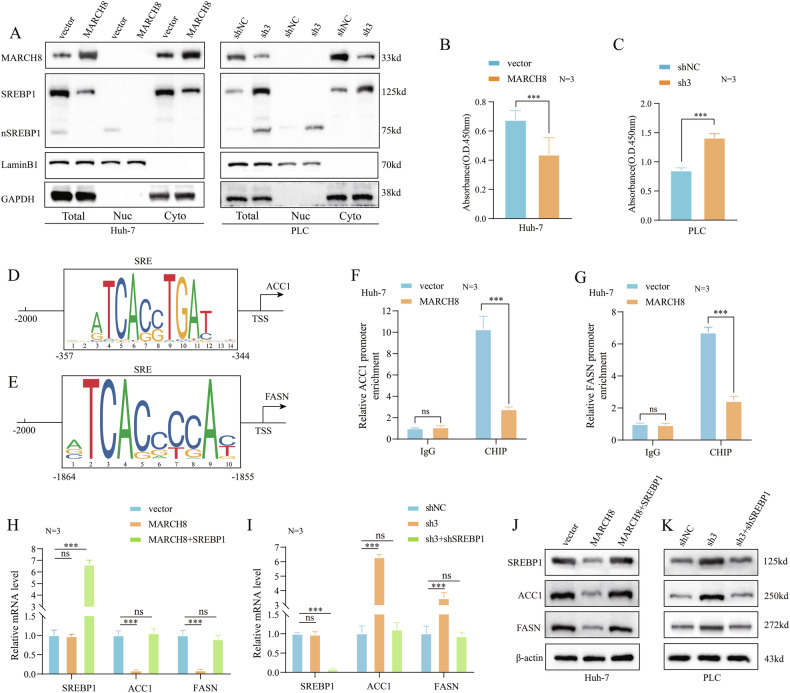
*MARCH8* inhibits the expression of *ACC1* and *FASN* by regulating *SREBP1.* **A** WB of *SREBP1*/n*SREBP1* in cytoplasmic and nuclear fractions of Huh7-*MARCH8* and PLC-sh3 cells. **B**, **C***SREBP1* DNA binding activity in Huh7-*MARCH8* and PLC-sh3 cells. **D**, **E***SREBP1* binding sites in *ACC1* and *FASN* promoters. **F**, **G** ChIP analysis of *SREBP1* binding to the *ACC1* and *FASN* promoters in Huh7-*MARCH8* cells and PLC-sh3 cells, and qPCR was conducted using primers specific to the *SREBP1* binding motifs. **H**–**K** qRT-PCR and WB of *SREBP1, ACC1,* and *FASN* expression in Huh7 and PLC cells with *MARCH8* and *SREBP1* co-overexpression or knockdown. ns: non-significant, ***: *p* < 0.001.

The transendoplasmic reticular membrane protein *SREBP1* functions as a transcription factor, promoting the transcription of genes that encode key enzymes in the FA synthesis pathway, such as *ACC1* and *FASN*. Afterward, it undergoes processing and maturation in the cytoplasm to produce nuclear sterol regulatory element-binding protein 1 n*SREBP1* [36]. The predominant hypothesis was that *MARCH8* reduces n*SREBP1* by degrading the *SREBP1* protein in the cytoplasm of HCC cells, thereby inhibiting the expression of *ACC1* and *FASN*. To substantiate this hypothesis, a cytoplasmic and nuclear fractionation assay was conducted, which revealed that *MARCH8* exerted a considerable effect on n*SREBP1* expression. The protein level of n*SREBP1* was markedly suppressed after overexpression of *MARCH8*, whereas the opposite pattern was observed when it was knocked down (Fig. 7A). In HCC cells, the transcriptional activity assay of *SREBP1* revealed that *MARCH8* substantially affects the DNA binding activity of *SREBP1* (Fig. 7B, C). Furthermore, *SREBP1* has the potential to directly bind to SRE, thereby activating the transcription of *ACC1* and *FASN*. To investigate this possibility, ChIP assays were performed in overexpress-*MARCH8* Huh7 cells, followed by qPCR of conserved *ACC1* and *FASN* promoter regions containing a consensus *SREBP1* binding SRE motif (Fig. 7D, E). This ChIP with the *SREBP1* antibody revealed that the *ACC1* and *FASN* promoter regions were more highly enriched in control cells than in Huh7 cells overexpressing *MARCH8* (Fig. 7F, G). These findings were consistent with the observation that the reconstitution of *SREBP1* in *MARCH8* modified HCC (overexpressed *MARCH8* Huh7 cells and *MARCH8* knockdown PLC cells) cells had a significant regulatory effect on the mRNA (Fig. 7H, I) and protein (Fig. 7J, K; Supplementary Fig. 5D, E) levels of *ACC1* and *FASN*.

### *MARCH8* suppresses HCC development by regulating *SREBP1*-mediated lipid accumulation

**Fig. 8:**
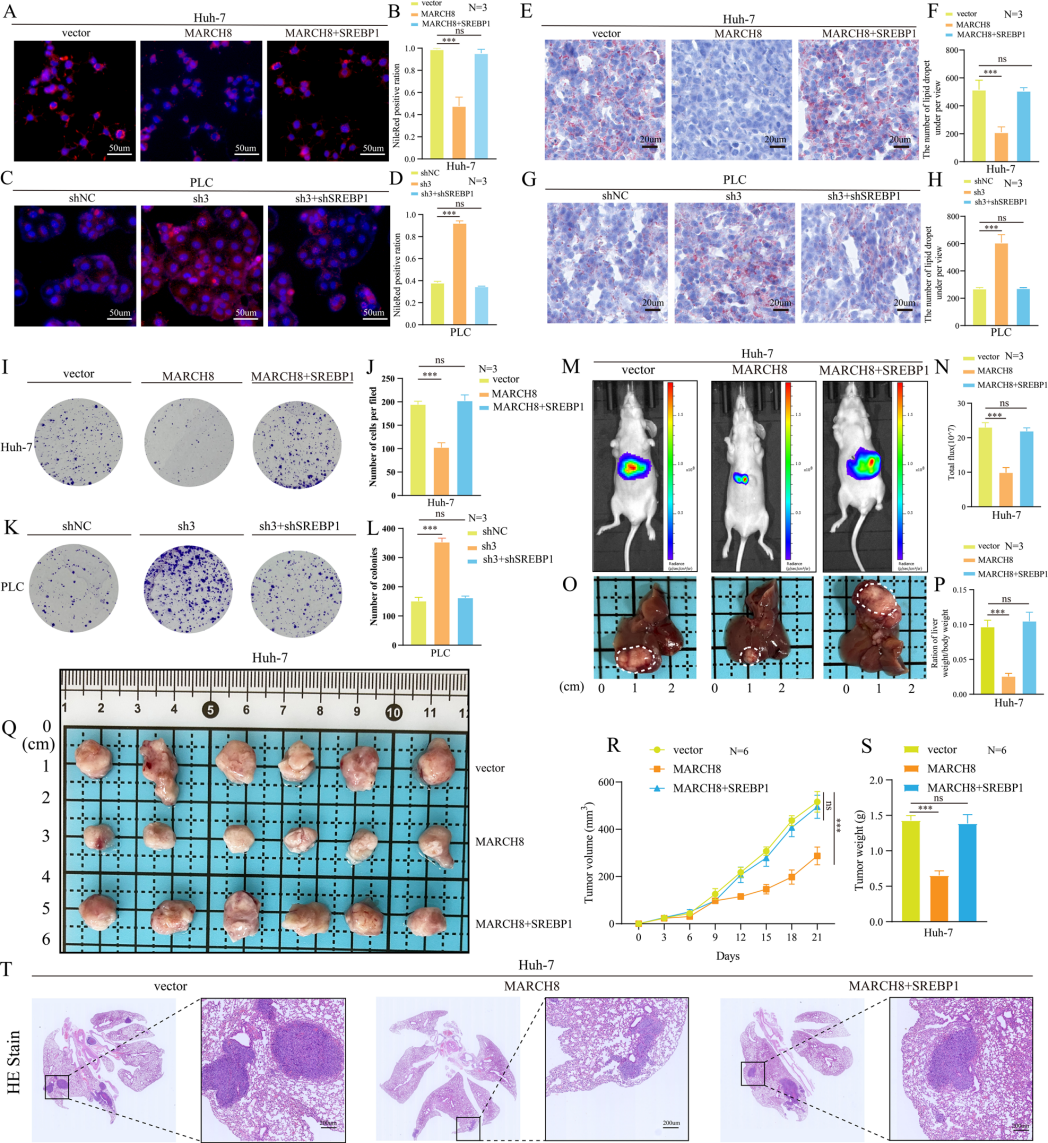
*MARCH8* is negatively associated with *SREBP1*-induced promotion of lipid accumulation and facilitation of HCC progression. **A**–**D** Nile Red and DAPI staining show lipid droplets (red) and nuclei (blue). **E**–**H** Oil Red O staining of lipid droplets in tumors. **I**–**L** Colony formation assay for cell proliferation. **M**, **N** In vivo, tumor growth was assessed by fluorescence imaging. **O**, **P** Tumor-to-body weight ratio in an orthotopic mouse model. **Q** Dissected subcutaneous tumors. **R**, **S** Tumor growth curve and weight analyses in nude mice. **T** H&E staining of lung sections. ns: non-significant, ***: *p* < 0.001.

Previous evidence suggests that *MARCH8* decreases the levels of *SREBP1* protein by increasing its ubiquitinated degradation and inhibiting the expression of crucial FA synthases. Based on this evidence, it was suggested that the reduction of *SREBP1* in HCC may be a factor in the *MARCH8*-mediated suppression of lipid accumulation and tumor progression. To validate this concept, we performed initial fluorescence microscopy and flow cytometry assessments of lipid droplet content in Huh7 and PLC cells under simultaneous co-downregulation or co-overexpression of *MARCH8* and *SREBP1*. The synchronous co-overexpression or co-knockdown of *MARCH8* and *SREBP1* yielded lipid droplet content similar to that of the control group (Fig. 8A–D; Supplementary Fig. 6A, B). Similarly, the Oil Red O staining demonstrated that the alterations in the quantity of lipid droplets in the mouse tumor tissues aligned with the outcomes of the Nile red staining (Fig. 8E–H). The findings suggest that the regulatory effect of *MARCH8* on lipids in HCC cells was predominantly dependent on *SREBP1*. Next, colony formation assays were performed, and found that the cells showed reduced and enhanced proliferative capacities after the overexpression and knockdown of *MARCH8*. However, simultaneously, the co-overexpression or co-knockdown of *MARCH8* and *SREBP1* did not yield a significant variation in cell proliferation compared to the control group (Fig. 8I–L). Moreover, an orthotopic liver tumor model was developed to evaluate the potential of the co-overexpression of *MARCH8* and *SREBP1* to revert the inhibition of tumor growth. As predicted, the orthotopic tumors’ sizes and mass showed no significant differences between the control group and those co-overexpressed of *MARCH8* and *SREBP1* (Fig. 8M–P). The nude mouse tumor-bearing model experiment demonstrates that the inhibitory effect of *MARCH8* overexpression on subcutaneous tumor formation in mice was reversed by the overexpression of *SREBP1* in Huh7 cells upregulated *MARCH8* expression (Fig. 8Q–S). Following the establishment of the xenograft model in nude mice, we performed WB analysis to validate the expression levels of *SREBP1*, *ACC1*, and *FASN* in subcutaneous tumors across three experimental groups: the vector group, the *MARCH8* overexpression group, and the co-overexpression group of *MARCH8* and *SREBP1*. The results were consistent with our previous findings, demonstrating that compared to the control group, *MARCH8* overexpression led to downregulation of *SREBP1*, *ACC1*, and *FASN* expression. Notably, this downregulation was reversed in the group co-overexpressing both *MARCH8* and *SREBP1*(Supplementary Fig. 6C–F). Furthermore, the overexpression of *SREBP1* can also restore the lung metastasis ability of Huh7 cells, which has been inhibited by the overexpression of *MARCH8* (Fig. 8T).

### *MARCH8*-*SREBP1* axis may play an important role in the prognosis and treatment of HCC

**Fig. 9:**
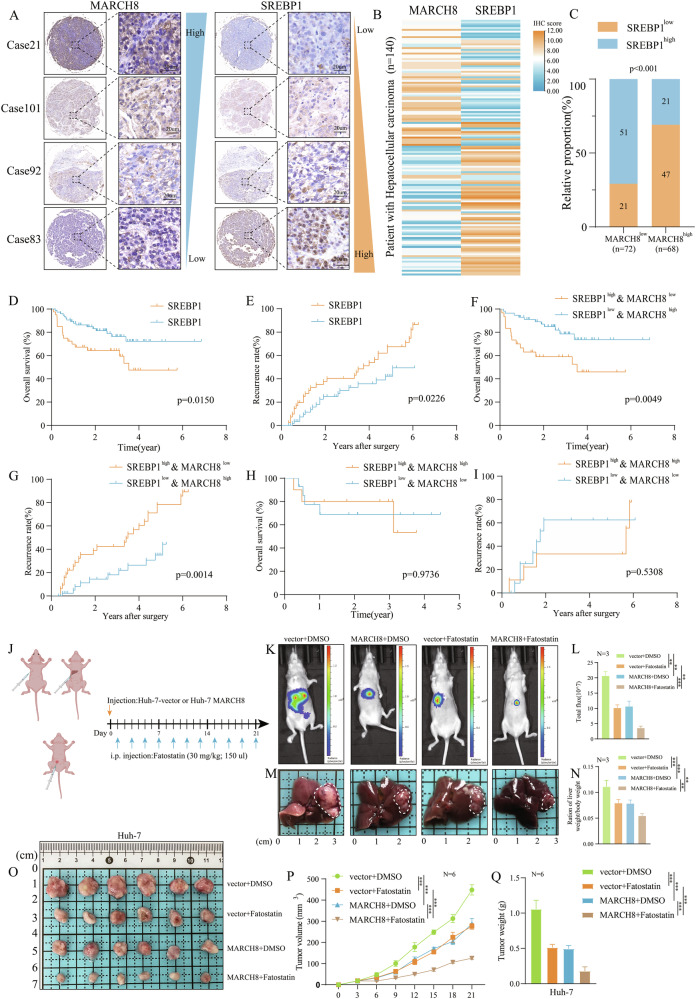
The *MARCH8*-*SREBP1* axis has potential clinical significance in the diagnosis and treatment of HCC. Representative IHC staining of *MARCH8* and *SREBP1* in HCC TMAs (**A**) and summarization into heat maps (**B**) and bar chart (**C**) according to H-score. **D**–**I** Kaplan–Meier analysis of the OS and RFS of *SREBP1*-positive vs. *SREBP1*-negative, *MARCH8*-positive & *SREBP1*-negative vs. *MARCH8*-negative & *SREBP1*-positive, and *MARCH8*-positive & *SREBP1-*positive vs. *MARCH8*-negative & *SREBP1*-negative patients based on HCC TMAs prognosis data. **J** Schematic showing the schedule of the mouse orthotopic liver tumor model, subcutaneous tumor models, and Fatostatin treatment. **K**, **L** A live imaging system assessed tumor growth in vivo by measuring fluorescence signals. **M**, **N** The ratio of orthotopic tumor to body weight of mice in the orthotopic model. Tumor images (**O**), growth curves (**P**), and weights (**Q**) were obtained on day 21 after dissection. **: *p* < 0.01, ***: *p* < 0.001.

IHC staining was performed on 140 HCC tissues to investigate the association between *MARCH8* and *SREBP1* expression in HCC tissues. The results indicated a negative correlation between the expression of *MARCH8* and *SREBP1* (Fig. 9A–C). The prognostic value of *MARCH8* and *SREBP1* in the HCC TMA dataset was then assessed collectively. It has been demonstrated that *SREBP1* plays a significant role in advancing HCC [37, 38]. As anticipated, the Kaplan–Meier survival analysis showed that the OS (Fig. 9D) and RFS rates (Fig. 9E) of patients with high *SREBP1* expression were inferior to those with low *SREBP1* expression. It was observed that in the current HCC TMA dataset, patients with elevated *MARCH8* levels and diminished *SREBP1* levels had markedly extended OS (Fig. 9F) and RFS rates (Fig. 9G) compared to those with reduced *MARCH8* levels and heightened *SREBP1* levels. However, no significant difference was observed in OS (Fig. 9H) or RFS rates (Fig. 9I) between patients with low *MARCH8* and *SREBP1* levels compared to patients with high *MARCH8* and *SREBP1* levels. It has been reported that Fatostatin, an *SREBP1* inhibitor, has the potential to impede tumor growth and activity [39, 40]. To determine whether *SREBP1* targeting inhibitors combined with *MARCH8* can also inhibit the progression of HCC in vivo, nude mouse orthotopic liver tumor models and subcutaneous tumor models were developed by injecting vector Huh7 cells or or *MARCH8*-overexpressing Huh7 cells (Fig. 9J). Interestingly, in the Huh7-vector group, treatment with the *SREBP1* inhibitor significantly suppressed tumor growth in orthotopic liver tumors (Fig. 9K–N) and subcutaneous tumors (Fig. 9O–Q) models. Moreover, the *SREBP1* inhibitor treatment significantly reduced tumor growth in the *MARCH8* Huh7 group. Overall, these experiments indicate that the *MARCH8*-*SREBP1* axis may play a significant role in the diagnosis, prognosis, and treatment of HCC, with implications for further research.

## Discussion

Cellular metabolism dysregulation is the hallmark of cancer [41]. Moreover, it has been demonstrated that HCC are associated with metabolic abnormalities [42]. Further, the rapid proliferation of HCC cells necessitates an increase in lipid synthesis and glycolysis [14, 43]. Furthermore, cancer metastasis invariably accompanies lipid metabolism alterations [44–46]. Consequently, further investigation into the aberrant lipid metabolism associated with HCC has considerable implications for developing targeted lipid metabolic interventions to impede the progression of HCC.

Recently, the E3 ubiquitin ligase family has been the focus of extensive research due to the potential role of oncoprotein stabilization in cancer progression [47, 48]. However, *MARCH8* has been shown to function as an antiviral factor [49], and its role in tumor progression has drawn more research attention. In breast cancer, *MARCH8* functions as a tumor suppressor by promoting the degradation of membrane and non-membrane proteins required for survival and metastasis [50]. It also shows tumor-suppressive activity in colorectal cancer (CRC) by regulating glycolysis [51]. Another study found that *MARCH8* was more highly expressed in HCC tissues than in adjacent non-cancerous tissues in 15 HCC patients and was associated with the progression of HCC [52]. However, this study yielded contradictory results by analyzing a larger clinical sample (140 samples), and revealed that the suppression of de novo FA synthesis is a critical mechanism by which *MARCH8* inhibits lipid accumulation and the progression of HCC. Further, *MARCH8*, an E3 ubiquitination ligase, typically binds to substrate proteins and enhances their ubiquitination-mediated proteasomal degradation. The binding protein of *MARCH8* was eventually obtained *via* co-IP and mass spectrometry. The outcomes showed that *SREBP1* is the main binding protein involved in the function of *MARCH8*. Although *MARCH8* appears to be highly expressed in HCC tissues than in normal tissues in the TCGA database [52], this does not contradict the current findings, as the TCGA database comprises transcriptome data from HCC patients rather than protein expression level data. This leads us to propose the hypothesis that a mechanism may restrict *MARCH8* protein expression, either by promoting *MARCH8* degradation or suppressing *MARCH8* translation. This mechanism necessitates further investigation.

*SREBP1*, an essential protein in the progression of HCC [53], is a key transcription factor for lipid synthesis that regulates lipid production by enhancing the transcription of critical enzymes such as *ACC1*, *ACLY*, *SCD1*, and *FASN* [29]. The results of this study demonstrate that *MARCH8* significantly inhibits lipid accumulation and HCC progression by suppressing the expression of *ACC1* and *FASN* through the K48-associated ubiquitination-mediated degradation of *SREBP1*. However, this study also showed that the TR2 domain of *MARCH8* can bind to the LC2 domain of *SREBP1*, which in turn promotes its ubiquitination and consequent degradation. However, further investigation is required to evaluate whether other *MARCH8* substrates are involved in lipid metabolic pathways.

The expression of *SREBP1* and *MARCH8* was found to be negatively correlated in HCC patient samples. Patients who showed low *MARCH8* and elevated *SREBP1* levels displayed poor OS. Moreover, this study revealed that *MARCH8* markedly suppressed the invasion and metastasis of HCC cells. The results indicate that *MARCH8-SREBP1* could function as a reliable prognostic marker and a potential treatment target. Furthermore, the *SREBP1* inhibitor was used in vitro to validate that its combination with *MARCH8* can more effectively impede the progression of HCC. Currently, there is a deficiency in the development of agonists or inhibitors for *MARCH8*, which have the potential to significantly impact the treatment of HCC. Further studies are necessary to understand the mechanisms that underlie the downregulation of *MARCH8* in HCC tissues and other potential regulatory pathways of *SREBP1*. The proposed approach may promote a comprehensive theoretical basis for targeting the *MARCH8-SREBP1* molecule as a potential therapeutic strategy for HCC.

Finally, hepatitis virus infection is frequently present in a significant number of patients with HCC, particularly in China. *MARCH8*, an antiviral factor [54], may also contribute to the inhibition of tumor progression by the antiviral effect in addition to its function of inhibiting FA synthesis in this study. This hypothesis needs further elaboration.

## Conclusions

This study concluded that *MARCH8* is a novel E3 ligase of *SREBP1*. The TR2 domain of *MARCH8* can bind to the LC2 domain of *SREBP1* and promote its ubiquitination, resulting in degradation *via* a K48-linked mechanism. This process reduces the transcriptional expression of downstream lipid metabolism-related genes, including *ACC1* and *FASN*. Therefore, the FA de novo synthesis process is modulated, and the progression of HCC is inhibited.

## Supplementary information

supplementary figure

Supplementary Table

Original Western blot

## Data Availability

The data supporting the findings of this study are available from the corresponding author upon reasonable request.

## References

[1] Villanueva A. Hepatocellular carcinoma. N Engl J Med. 2019;380:1450–62. 30970190 10.1056/NEJMra1713263

[2] Llovet JM, Kelley RK, Villanueva A, Singal AG, Pikarsky E, Roayaie S, et al. Hepatocellular carcinoma. Nat Rev Dis Prim. 2021;7:6. 33479224 10.1038/s41572-020-00240-3 PMC13537433

[3] Jun BG, Kim M, Shin HS, Yi JJ, Yi SW. Impact of overweight and obesity on the risk of hepatocellular carcinoma: a prospective cohort study in 14.3 million Koreans. Br J Cancer. 2022;127:109–15. 35249102 10.1038/s41416-022-01771-0 PMC9276765

[4] El-Serag HB. Hepatocellular carcinoma. N Engl J Med. 2011;365:1118–27. 21992124 10.1056/NEJMra1001683

[5] Fujiwara N, Nakagawa H, Kudo Y, Tateishi R, Taguri M, Watadani T, et al. Sarcopenia, intramuscular fat deposition, and visceral adiposity independently predict the outcomes of hepatocellular carcinoma. J Hepatol. 2015;63:131–40. 25724366 10.1016/j.jhep.2015.02.031

[6] Park EJ, Lee JH, Yu GY, He G, Ali SR, Holzer RG, et al. Dietary and genetic obesity promote liver inflammation and tumorigenesis by enhancing IL-6 and TNF expression. Cell. 2010;140:197–208. 20141834 10.1016/j.cell.2009.12.052 PMC2836922

[7] Tilg H, Hotamisligil GS. Nonalcoholic fatty liver disease: cytokine-adipokine interplay and regulation of insulin resistance. Gastroenterology. 2006;131:934–45. 16952562 10.1053/j.gastro.2006.05.054

[8] Nakagawa H, Umemura A, Taniguchi K, Font-Burgada J, Dhar D, Ogata H, et al. ER stress cooperates with hypernutrition to trigger TNF-dependent spontaneous HCC development. Cancer Cell. 2014;26:331–43. 25132496 10.1016/j.ccr.2014.07.001 PMC4165611

[9] Yoshimoto S, Loo TM, Atarashi K, Kanda H, Sato S, Oyadomari S, et al. Obesity-induced gut microbial metabolite promotes liver cancer through senescence secretome. Nature. 2013;499:97–101. 23803760 10.1038/nature12347

[10] Nakagawa H. Recent advances in mouse models of obesity- and nonalcoholic steatohepatitis-associated hepatocarcinogenesis. World J Hepatol. 2015;7:2110–8. 26301053 10.4254/wjh.v7.i17.2110 PMC4539404

[11] Ward PS, Thompson CB. Metabolic reprogramming: a cancer hallmark even Warburg did not anticipate. Cancer Cell. 2012;21:297–308. 22439925 10.1016/j.ccr.2012.02.014 PMC3311998

[12] Currie E, Schulze A, Zechner R, Walther TC, Farese RV Jr. Cellular fatty acid metabolism and cancer. Cell Metab. 2013;18:153–61. 23791484 10.1016/j.cmet.2013.05.017 PMC3742569

[13] Muir K, Hazim A, He Y, Peyressatre M, Kim DY, Song X, et al. Proteomic and lipidomic signatures of lipid metabolism in NASH-associated hepatocellular carcinoma. Cancer Res. 2013;73:4722–31. 23749645 10.1158/0008-5472.CAN-12-3797 PMC3855016

[14] Pope ED 3rd, Kimbrough EO, Vemireddy LP, Surapaneni PK, Copland JA 3rd, Mody K. Aberrant lipid metabolism as a therapeutic target in liver cancer. Expert Opin Ther Targets. 2019;23:473–83. 31076001 10.1080/14728222.2019.1615883 PMC6594827

[15] Budhu A, Roessler S, Zhao X, Yu Z, Forgues M, Ji J, et al. Integrated metabolite and gene expression profiles identify lipid biomarkers associated with progression of hepatocellular carcinoma and patient outcomes. Gastroenterology. 2013;144:1066–75.e1. 23376425 10.1053/j.gastro.2013.01.054 PMC3633738

[16] Rysman E, Brusselmans K, Scheys K, Timmermans L, Derua R, Munck S, et al. De novo lipogenesis protects cancer cells from free radicals and chemotherapeutics by promoting membrane lipid saturation. Cancer Res. 2010;70:8117–26. 20876798 10.1158/0008-5472.CAN-09-3871

[17] Bergers G, Fendt SM. The metabolism of cancer cells during metastasis. Nat Rev Cancer. 2021;21:162–80. 33462499 10.1038/s41568-020-00320-2 PMC8733955

[18] Velázquez AP, Graef M. Autophagy regulation depends on ER homeostasis controlled by lipid droplets. Autophagy. 2016;12:1409–10. 27245853 10.1080/15548627.2016.1190074 PMC4968306

[19] Cao J, Tu DY, Zhou J, Jiang GQ, Jin SJ, Su BB, et al. Comprehensive analysis of the clinical significance, immune infiltration, and biological role of MARCH ligases in HCC. Front Immunol. 2022;13:997265. 36263042 10.3389/fimmu.2022.997265 PMC9573977

[20] Senft D, Qi J, Ronai ZA. Ubiquitin ligases in oncogenic transformation and cancer therapy. Nat Rev Cancer. 2018;18:69–88. 29242641 10.1038/nrc.2017.105 PMC6054770

[21] Zhou J, Tu D, Peng R, Tang Y, Deng Q, Su B, et al. RNF173 suppresses RAF/MEK/ERK signaling to regulate invasion and metastasis via GRB2 ubiquitination in hepatocellular carcinoma. Cell Commun Signal. 2023;21:224. 37626338 10.1186/s12964-023-01241-x PMC10464048

[22] Lin H, Feng L, Cui KS, Zeng LW, Gao D, Zhang LX, et al. The membrane-associated E3 ubiquitin ligase MARCH3 downregulates the IL-6 receptor and suppresses colitis-associated carcinogenesis. Cell Mol Immunol. 2021;18:2648–59. 34785732 10.1038/s41423-021-00799-1 PMC8632971

[23] Bartee E, Mansouri M, Hovey Nerenberg BT, Gouveia K, Früh K. Downregulation of major histocompatibility complex class I by human ubiquitin ligases related to viral immune evasion proteins. J Virol. 2004;78:1109–20. 14722266 10.1128/JVI.78.3.1109-1120.2004 PMC321412

[24] Tada T, Zhang Y, Koyama T, Tobiume M, Tsunetsugu-Yokota Y, Yamaoka S, et al. MARCH8 inhibits HIV-1 infection by reducing virion incorporation of envelope glycoproteins. Nat Med. 2015;21:1502–7. 26523972 10.1038/nm.3956

[25] van de Kooij B, Verbrugge I, de Vries E, Gijsen M, Montserrat V, Maas C, et al. Ubiquitination by the membrane-associated RING-CH-8 (MARCH-8) ligase controls steady-state cell surface expression of tumor necrosis factor-related apoptosis inducing ligand (TRAIL) receptor 1. J Biol Chem. 2013;288:6617–28. 23300075 10.1074/jbc.M112.448209 PMC3585101

[26] Singh S, Saraya A, Das P, Sharma R. Increased expression of MARCH8, an E3 ubiquitin ligase, is associated with growth of esophageal tumor. Cancer Cell Int. 2017;17:116. 29213217 10.1186/s12935-017-0490-y PMC5715508

[27] Kim MH, Rebbert ML, Ro H, Won M, Dawid IB. Cell adhesion in zebrafish embryos is modulated by March 8. PLoS ONE. 2014;9:e94873. 24752240 10.1371/journal.pone.0094873 PMC3994051

[28] Shimano H, Sato R. SREBP-regulated lipid metabolism: convergent physiology - divergent pathophysiology. Nat Rev Endocrinol. 2017;13:710–30. 28849786 10.1038/nrendo.2017.91

[29] Horton JD, Goldstein JL, Brown MS. SREBPs: activators of the complete program of cholesterol and fatty acid synthesis in the liver. J Clin Investig. 2002;109:1125–31. 11994399 10.1172/JCI15593 PMC150968

[30] Düvel K, Yecies JL, Menon S, Raman P, Lipovsky AI, Souza AL, et al. Activation of a metabolic gene regulatory network downstream of mTOR complex 1. Mol Cell. 2010;39:171–83. 20670887 10.1016/j.molcel.2010.06.022 PMC2946786

[31] Ruiz R, Jideonwo V, Ahn M, Surendran S, Tagliabracci VS, Hou Y, et al. Sterol regulatory element-binding protein-1 (SREBP-1) is required to regulate glycogen synthesis and gluconeogenic gene expression in mouse liver. J Biol Chem. 2014;289:5510–7. 24398675 10.1074/jbc.M113.541110 PMC3937627

[32] Wang H, Humbatova A, Liu Y, Qin W, Lee M, Cesarato N, et al. Mutations in SREBF1, encoding sterol regulatory element binding transcription factor 1, cause autosomal-dominant IFAP syndrome. Am J Hum Genet. 2020;107:34–45. 32497488 10.1016/j.ajhg.2020.05.006 PMC7332643

[33] Ma APY, Yeung CLS, Tey SK, Mao X, Wong SWK, Ng TH, et al. Suppression of ACADM-mediated fatty acid oxidation promotes hepatocellular carcinoma via aberrant CAV1/SREBP1 signaling. Cancer Res. 2021;81:3679–92. 33975883 10.1158/0008-5472.CAN-20-3944

[34] Zoller H, Tilg H. Nonalcoholic fatty liver disease and hepatocellular carcinoma. Metabolism. 2016;65:1151–60. 26907206 10.1016/j.metabol.2016.01.010

[35] Akimov V, Barrio-Hernandez I, Hansen SVF, Hallenborg P, Pedersen AK, Bekker-Jensen DB, et al. UbiSite approach for comprehensive mapping of lysine and N-terminal ubiquitination sites. Nat Struct Mol Biol. 2018;25:631–40. 29967540 10.1038/s41594-018-0084-y

[36] Han J, Li E, Chen L, Zhang Y, Wei F, Liu J, et al. The CREB coactivator CRTC2 controls hepatic lipid metabolism by regulating SREBP1. Nature. 2015;524:243–6. 26147081 10.1038/nature14557

[37] Wang C, Tong Y, Wen Y, Cai J, Guo H, Huang L, et al. Hepatocellular carcinoma-associated protein TD26 interacts and enhances sterol regulatory element-binding protein 1 activity to promote tumor cell proliferation and growth. Hepatology. 2018;68:1833–50. 29663480 10.1002/hep.30030

[38] Chen J, Ding C, Chen Y, Hu W, Yu C, Peng C, et al. ACSL4 reprograms fatty acid metabolism in hepatocellular carcinoma via c-Myc/SREBP1 pathway. Cancer Lett. 2021;502:154–65. 33340617 10.1016/j.canlet.2020.12.019

[39] Talebi A, Dehairs J, Rambow F, Rogiers A, Nittner D, Derua R, et al. Sustained SREBP-1-dependent lipogenesis as a key mediator of resistance to BRAF-targeted therapy. Nat Commun. 2018;9:2500. 29950559 10.1038/s41467-018-04664-0 PMC6021375

[40] Cao Y, Wang X, Liu Y, Liu P, Qin J, Zhu Y, et al. BHLHE40 inhibits ferroptosis in pancreatic cancer cells via upregulating SREBF1. Adv Sci. 2024;11:e2306298. 10.1002/advs.202306298 PMC10870036 38064101

[41] Hanahan D, Weinberg RA. Hallmarks of cancer: the next generation. Cell. 2011;144:646–74. 21376230 10.1016/j.cell.2011.02.013

[42] Anstee QM, Reeves HL, Kotsiliti E, Govaere O, Heikenwalder M. From NASH to HCC: current concepts and future challenges. Nat Rev Gastroenterol Hepatol. 2019;16:411–28. 31028350 10.1038/s41575-019-0145-7

[43] Cheng X, Geng F, Pan M, Wu X, Zhong Y, Wang C, et al. Targeting DGAT1 ameliorates glioblastoma by increasing fat catabolism and oxidative stress. Cell Metab. 2020;32:229–42.e8. 32559414 10.1016/j.cmet.2020.06.002 PMC7415721

[44] Hanahan D. Hallmarks of cancer: new dimensions. Cancer Discov. 2022;12:31–46. 35022204 10.1158/2159-8290.CD-21-1059

[45] Bian X, Liu R, Meng Y, Xing D, Xu D, Lu Z. Lipid metabolism and cancer. J Exp Med. 2021;218:e20201606. 33601415 10.1084/jem.20201606 PMC7754673

[46] Carracedo A, Cantley LC, Pandolfi PP. Cancer metabolism: fatty acid oxidation in the limelight. Nat Rev Cancer. 2013;13:227–32. 23446547 10.1038/nrc3483 PMC3766957

[47] Wang H, Yang W, Qin Q, Yang X, Yang Y, Liu H, et al. E3 ubiquitin ligase MAGI3 degrades c-Myc and acts as a predictor for chemotherapy response in colorectal cancer. Mol Cancer. 2022;21:151. 35864508 10.1186/s12943-022-01622-9 PMC9306183

[48] Jiang Q, Li F, Cheng Z, Kong Y, Chen C. The role of E3 ubiquitin ligase HECTD3 in cancer and beyond. Cell Mol Life Sci. 2020;77:1483–95. 31637449 10.1007/s00018-019-03339-3 PMC11105068

[49] Villalón-Letelier F, Brooks AG, Londrigan SL, Reading PC. MARCH8 restricts influenza A virus infectivity but does not downregulate viral glycoprotein expression at the surface of infected cells. mBio. 2021;12:e0148421. 34517760 10.1128/mBio.01484-21 PMC8546552

[50] Chen W, Patel D, Jia Y, Yu Z, Liu X, Shi H, et al. MARCH8 suppresses tumor metastasis and mediates degradation of STAT3 and CD44 in breast cancer cells. Cancers. 2021;13:2550. 34067416 10.3390/cancers13112550 PMC8196951

[51] Wang Z, Wang MM, Geng Y, Ye CY, Zang YS. Membrane-associated RING-CH protein (MARCH8) is a novel glycolysis repressor targeted by miR-32 in colorectal cancer. J Transl Med. 2022;20:402. 36064706 10.1186/s12967-022-03608-z PMC9446774

[52] Xu Y, Zhang D, Ji J, Zhang L. Ubiquitin ligase MARCH8 promotes the malignant progression of hepatocellular carcinoma through PTEN ubiquitination and degradation. Mol Carcinog. 2023;62:1062–72. 37098835 10.1002/mc.23546

[53] Yamashita T, Honda M, Takatori H, Nishino R, Minato H, Takamura H, et al. Activation of lipogenic pathway correlates with cell proliferation and poor prognosis in hepatocellular carcinoma. J Hepatol. 2009;50:100–10. 19008011 10.1016/j.jhep.2008.07.036

[54] Liu X, Xu F, Ren L, Zhao F, Huang Y, Wei L, et al. MARCH8 inhibits influenza A virus infection by targeting viral M2 protein for ubiquitination-dependent degradation in lysosomes. Nat Commun. 2021;12:4427. 34285233 10.1038/s41467-021-24724-2 PMC8292393

