# Supplementary material for: MARCH8 suppresses hepatocellular carcinoma by promoting SREBP1 degradation and modulating fatty acid de novo synthesis
Source: Cell Death Dis. 2025 May 16;16(1):391. doi: 10.1038/s41419-025-07707-9 (PMC12084374; doi:10.1038/s41419-025-07707-9)
Supplement: Supplementary file 1 — supplementary figure [file 41419_2025_7707_MOESM1_ESM.docx]

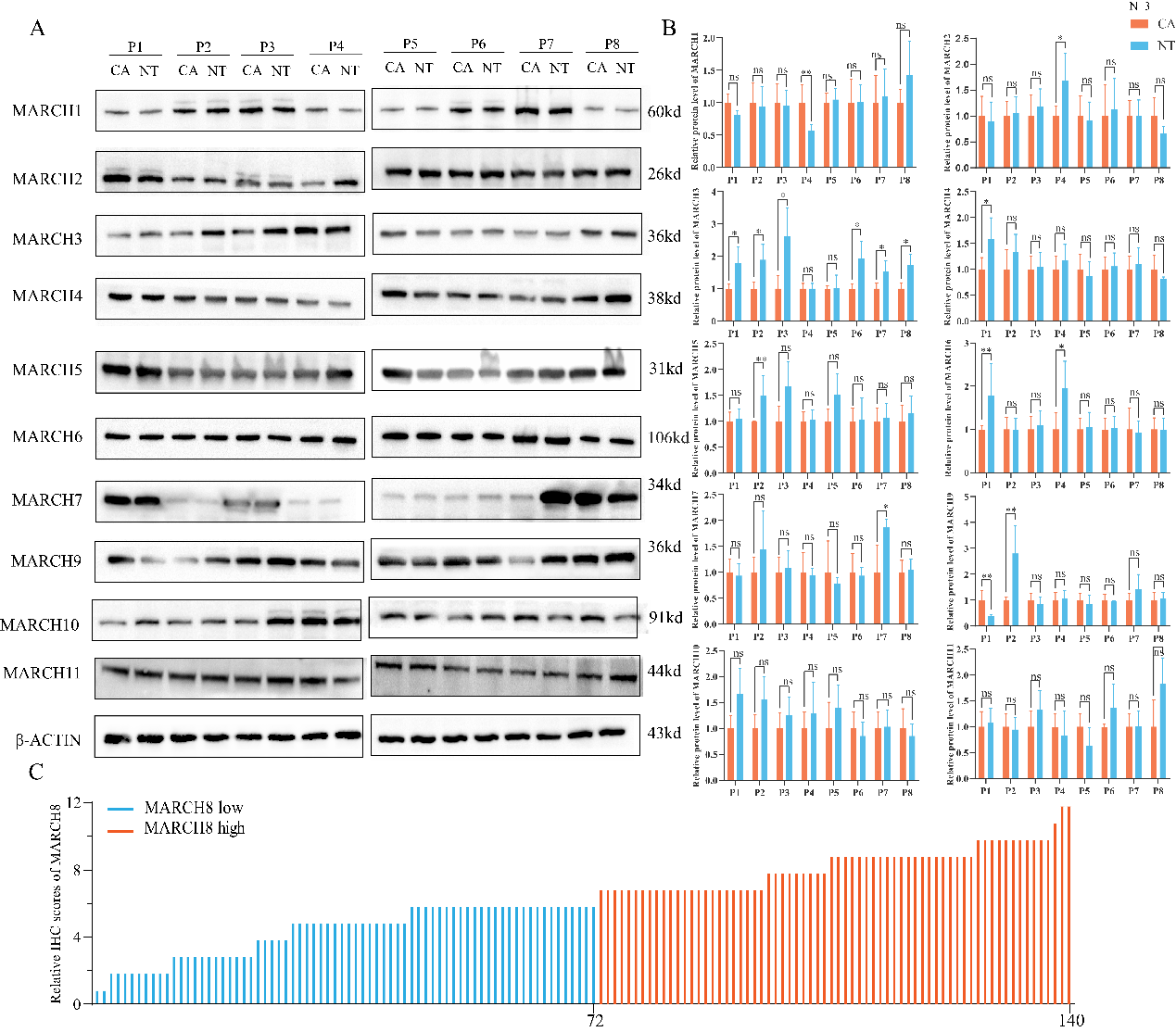
Supp Fig 1. (A, B) WB of *MARCH* ligases were performed on 8 pairs of HCC cancer tissues and matched non-tumor tissues. (C) *MARCH8* low-expression group comprised 72 patients, and the high-expression group included 68 patients, based on the H-score. ns: non-significant, *: *p* < 0.05, **: *p* < 0.01.

#
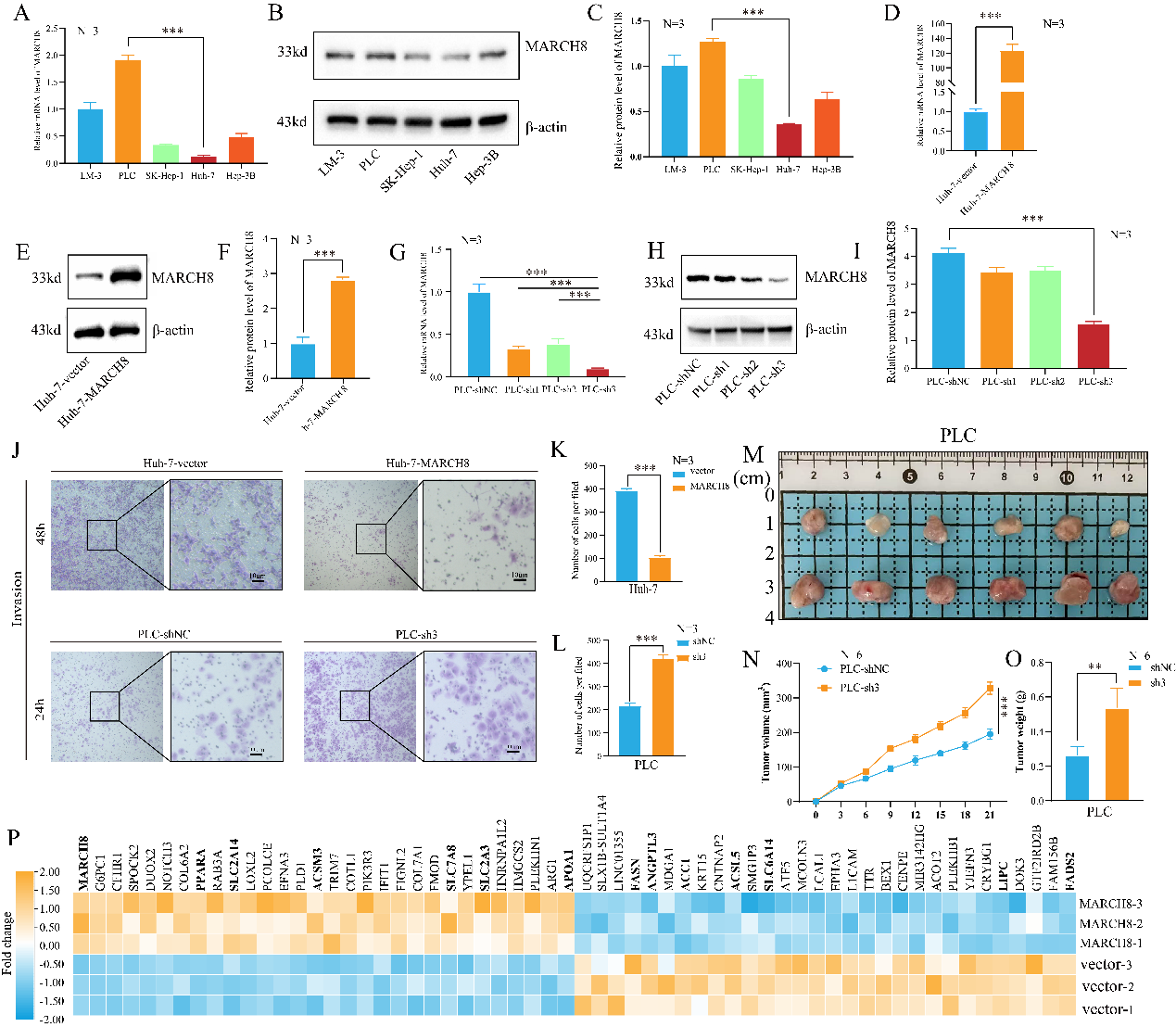
Supp Fig 2. (A-C) Expression of mRNA and protein of *MARCH8* in different cell lines. WB and qRT-PCR confirmed the efficiency of *MARCH8* cDNA lentivirus transfection in Huh7 cell lines (D-F) and the *MARCH8*-shRNA lentivirus (sh1, sh2, and sh3) in PLC cell lines(G-I). (J-L) Effect of *MARCH8* on cell invasion via transwell assays. PLC-shNC or PLC-sh3 cells were injected into the dorsal subcutis or liver parenchyma of nude mice. Subcutaneous tumor volumes were measured every 3 days. Tumor images (M), growth curves (N) and weights (O) were obtained on day 21 after dissection. (P)Heatmap depicting the top 30 upregulated and downregulated genes in Huh7 cell transduced with *MARCH8* cDNA vector lentivirus. **: *p* < 0.01, ***: *p* < 0.001.

#
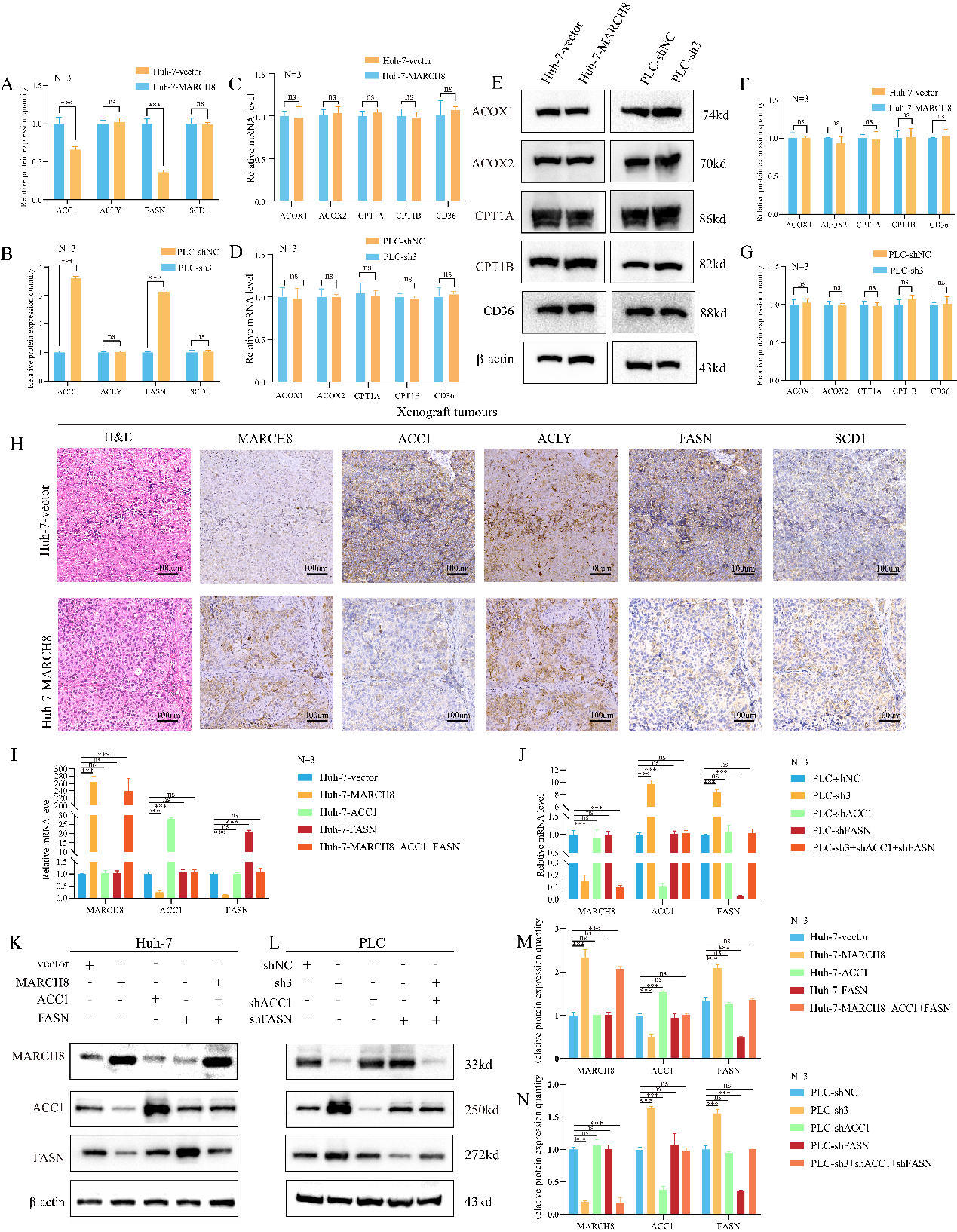
Supp Fig 3. (A) Quantification of WB Detected *ACC1*, *ACLY*, *FASN* and *SCD1* Protein Levels in Huh7-vector and Huh7-*MARCH8* cells*.* (B) Quantification of WB Detected *ACC1*, *ACLY*, *FASN* and *SCD1* Protein Levels in PLC-shNC and PLC-sh3 cells*.* Analysis of *ACOX1*, *ACOX2*, *CPT1A*, *CPT1B* and *CD36* in Huh7-vector and Huh7-*MARCH8 or* PLC-shNC and PLC-sh3 cells by qRT-PCR (C, D) and Western blot (E-G). (H) IHC staining showed the expression of *MARCH8*, *ACC1* and *FASN* in the subcutaneous tumor tissues of mice with Huh7-vector and Huh7-*MARCH8* group. (I, J) qRT-PCR experiments to verify mRNA expression levels after transfection with co-overexpressed or co-knockdown *ACC1* and *FASN* lentiviruses. (K-N) WB experiments to verify protein expression levels after transfection with co-overexpressed or co-knockdown *ACC1* and *FASN* lentiviruses. ns: non-significant, ***: *p* < 0.001.

#
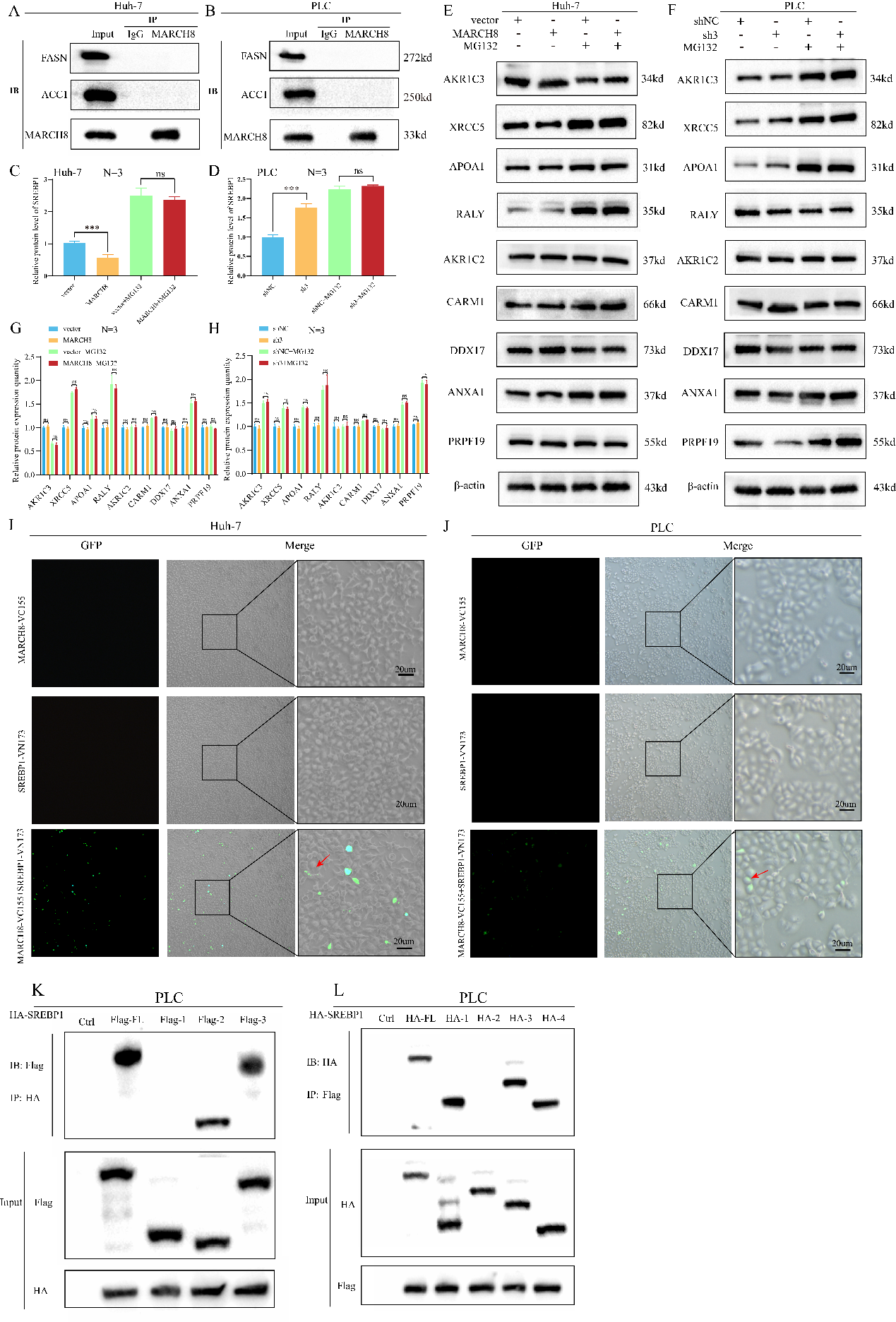
Supp Fig 4. (A, B) Huh7 and PLC cell lysates were immunoprecipitated with IgG or *MARCH8* antibodies and immunoblotted with *ACC1* or *FASN* antibodies. Quantification of SREBP1 Levels Detected by WB in Huh7-*MARCH8*(C) and PLC-sh3(D) cells Following Treatment with MG132 (10 µM for 6 Hours). Western blot analysis of lipid metabolism-related protein levels in Huh7- *MARCH8* (E, G) and PLC-sh*3* (F, H) cells in the presence of MG132(10 uM) treatment for 6 hours. (I, J) BiFC technique was utilized to further examine the subcellular localization of the interaction in Huh7 (I) and PLC cells (J). (K, L) Co-IP assays clarify specific binding domains of *MARCH8* and *SREBP1* in PLC cells. ns: non-significant, ***: *p* < 0.001.

#
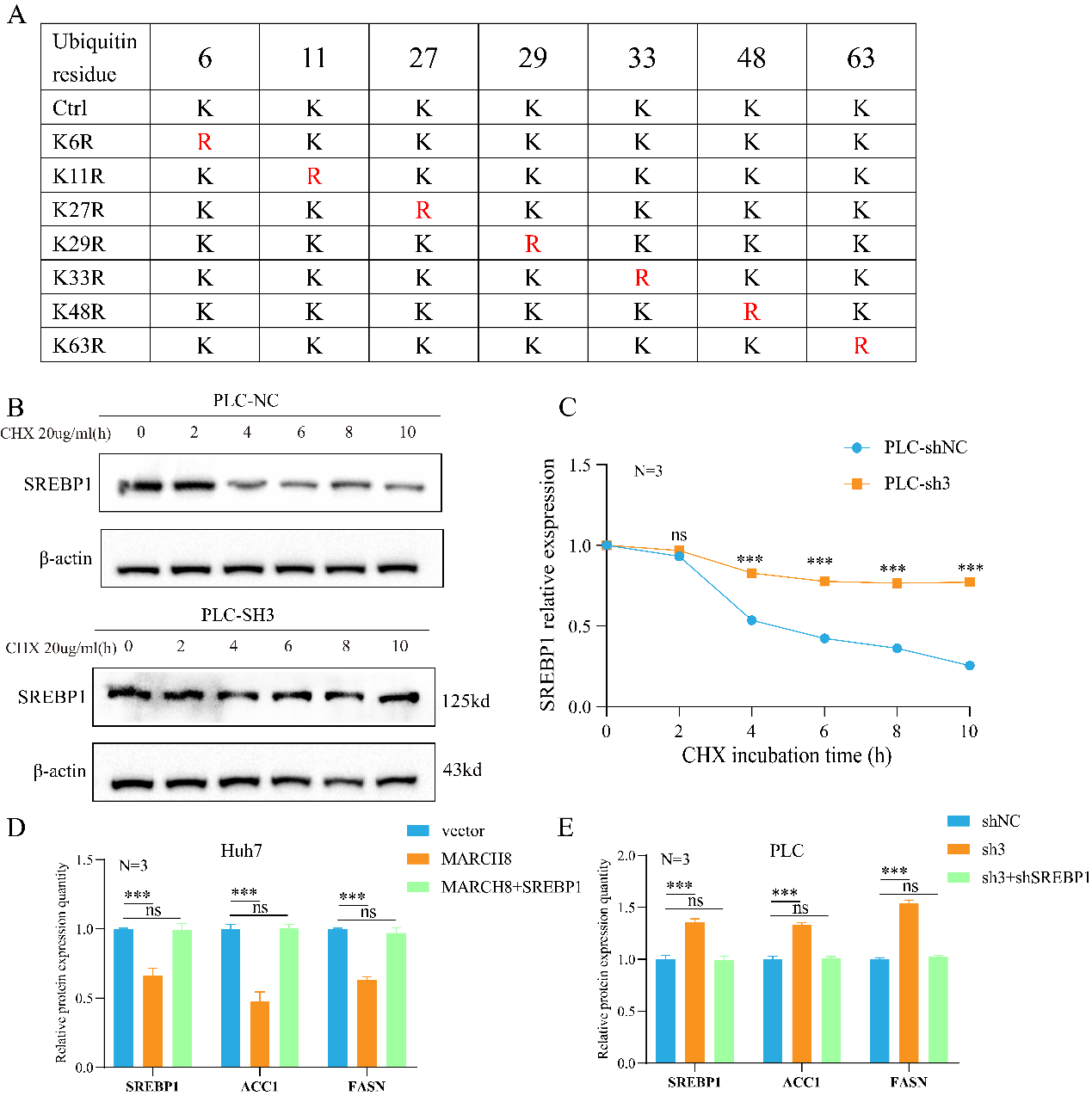
Supp Fig 5. (A)Schematic diagram of a ubiquitinating plasmid and its mutants. (B, C) Western blot showing the decrease of *SREBP1* protein at the indicated time points after the addition of cycloheximide (20 µg/ml) to the PLC cells. (D) Quantification of WB Detected *SREBP1*, *ACC1* and *FASN* Protein Levels in Huh7-vector, Huh7-*MARCH8* and Huh7- *MARCH8+SREBP1*cells. (E) Quantification of WB Detected *SREBP1*, *ACC1* and *FASN* Protein Levels in PLC-shNC, PLC-sh3 and PLC-sh3*+shSREBP1*cells. ns: non-significant, ***: *p* < 0.001.

#
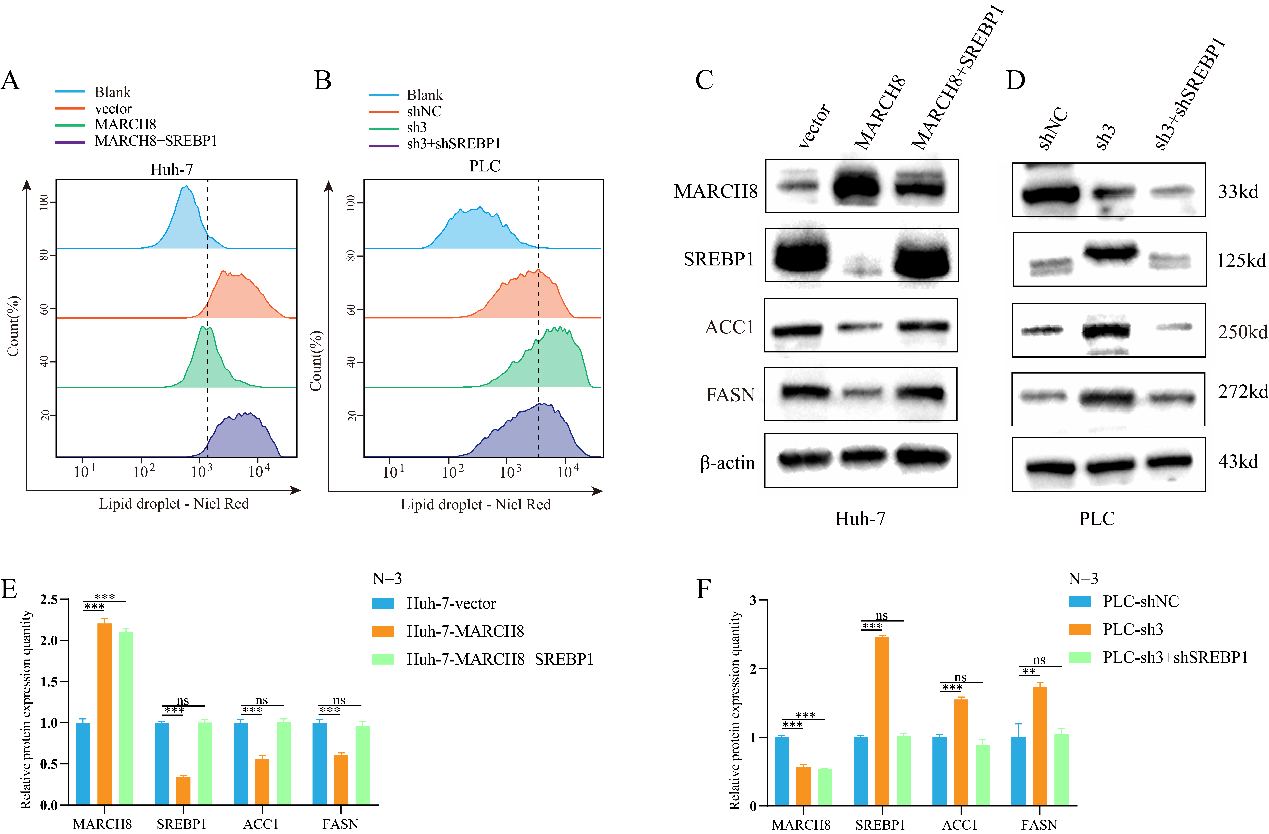
Supp Fig 6. (A, B) The fluorescence intensity of the Nile Red staining was determined by flow cytometry. (C-F) The expression levels of SREBP1, ACC1, and FASN at the animal level through Western blot (WB) experiments were evaluated after reconstructing the xenograft model in nude mice. ns: non-significant, **: *p* < 0.01, ***: *p* < 0.001.
