# Supplementary material for: MARCH8 suppresses hepatocellular carcinoma by promoting SREBP1 degradation and modulating fatty acid de novo synthesis
Source: Cell Death Dis. 2025 May 16;16(1):391. doi: 10.1038/s41419-025-07707-9 (PMC12084374; doi:10.1038/s41419-025-07707-9)
Supplement: Supplementary file 2 — Supplementary Table [file 41419_2025_7707_MOESM2_ESM.docx]

Supplementary Table1

| Antibody | RESOURCE | IDENTIFIER |
| --- | --- | --- |
| Rabbit anti-MARCH1 | Proteintech | Cat# 24771-1-AP |
| Rabbit anti-MARCH2 | ThermoFisher | Cat# PA5-30620 |
| Rabbit anti-MARCH3 | ThermoFisher | Cat# PA5-60351 |
| Rabbit anti-MARCH4 | ThermoFisher | Cat# PA5-116198 |
| Rabbit anti-MARCH5 | ThermoFisher | Cat# PA5-25584 |
| Mouse anti-MARCH6 | Santa Cruz | Cat# sc-517051 |
| Mouse anti-MARCH7 | Sigma | Cat# PA5-54572 |
| Rabbit anti-MARCH8 | Proteintech | Cat# 14119-1-AP |
| Rabbit anti-MARCH9 | ThermoFisher | Cat# PA5-103817 |
| Rabbit anti-MARCH10 | ThermoFisher | Cat# PA5-116260 |
| Rabbit anti-MARCH11 | ThermoFisher | Cat# PA5-69239 |
| Mouse anti-β-actin | Proteintech | Cat# 60008-1-Ig |
| Rabbit anti-ACC1 | Proteintech | Cat# 21923-1-AP |
| Mouse anti- FASN | Proteintech | Cat# 66591-1-Ig |
| Rabbit anti-ACLY | Proteintech | Cat# 15421-1-AP |
| Rabbit anti-SCD1 | Proteintech | Cat# 28678-1-AP |
| Rabbit anti-CD26 | Proteintech | Cat# 18836-1-AP |
| Rabbit anti-ACOX1 | Proteintech | Cat# 10957-1-AP |
| Rabbit anti-ACOX2 | Proteintech | Cat# 17571-1-AP |
| Mouse anti-CPT1A | Proteintech | Cat# 66039-1-Ig |
| Rabbit anti-CPT1B | Proteintech | Cat# 22170-1-AP |
| Rabbit anti-SREBF1 | Proteintech | Cat# 14088-1-AP |
| Mouse anti-Ubiquitin | Santa Cruz | Cat# sc-8017 |
| Rabbit anti-FLAG | Proteintech | Cat# 20543-1-AP |
| Rabbit anti-HA | Proteintech | Cat# 51064-2-AP |
| Mouse anti-MYC | Proteintech | Cat# 60003-2-Ig |
| Rabbit anti-LaminB1 | Proteintech | Cat# 12987-1-AP |
| Rabbit anti-GAPDH | Proteintech | Cat# 10494-1-AP |

Supplementary Table2

| Primer name | Primer sequence (5'to3') |
| --- | --- |
| MARCH8-F | GGAGAGGGAAGAACAGAATGAGAAGAC |
| MARCH8-R | GATGGCGTGATAGAAGTGCGAGAG |
| β-actin-F | CATGTACGTTGCTATCCAGGC |
| β-actin-R | CTCCTTAATGTCACGCACGAT |
| ACC1-F | ATGTCTGGCTTGCACCTAGTA |
| ACC1-R | CCCCAAAGCGAGTAACAAATTCT |
| ACLY-F | TCGGCCAAGGCAATTTCAGAG |
| ACLY-R | CGAGCATACTTGAACCGATTCT |
| FASN-F | AAGGACCTGTCTAGGTTTGATGC |
| FASN-R | TGGCTTCATAGGTGACTTCCA |
| SCD1-F | TCTAGCTCCTATACCACCACCA |
| SCD1-R | TCGTCTCCAACTTATCTCCTCC |
| ACOX1-F | TGCTCAGAAAGAGAAATGGC |
| ACOX1-R | TGGGTTTCAGGGTCATACG |
| ACOX2-F | GCACCCCGACATAGAGAGC |
| ACOX2-R | CTGCGGAGTGCAGTGTTCT |
| CD36-F | GGCTGTGACCGGAACTGTG |
| CD36-R | AGGTCTCCAACTGGCATTAGAA |
| SREBP1-F | AGGTCTCCAACTGGCATTAGAA |
| SREBP1-R | GCATGGACGGGTACATCTTCAA |

Supplementary Table3

ACC1 and FASN specific primer sequences in chip experiment

| Primer name | Primer sequence (5'to3') |
| --- | --- |
| ACC1-F | CATGCGGTCTATCCGTAGGTG |
| ACC1-R | GTGTGACCATGACAACGAATCT |
| FASN -F | CCGAGACACTCGTGGGCTA |
| FASN -R | CTTCAGCAGGACATTGATGCC |
