# Supplementary material for: MARCH8 suppresses hepatocellular carcinoma by promoting SREBP1 degradation and modulating fatty acid de novo synthesis
Source: Cell Death Dis. 2025 May 16;16(1):391. doi: 10.1038/s41419-025-07707-9 (PMC12084374; doi:10.1038/s41419-025-07707-9)

**Original Western blot**

**Fig 1**

B

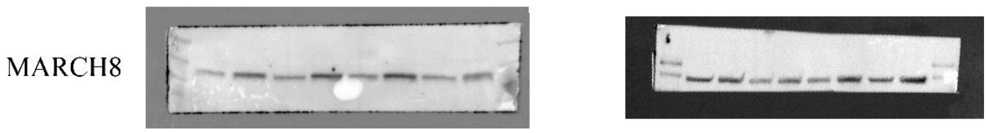


**Fig 4**

B

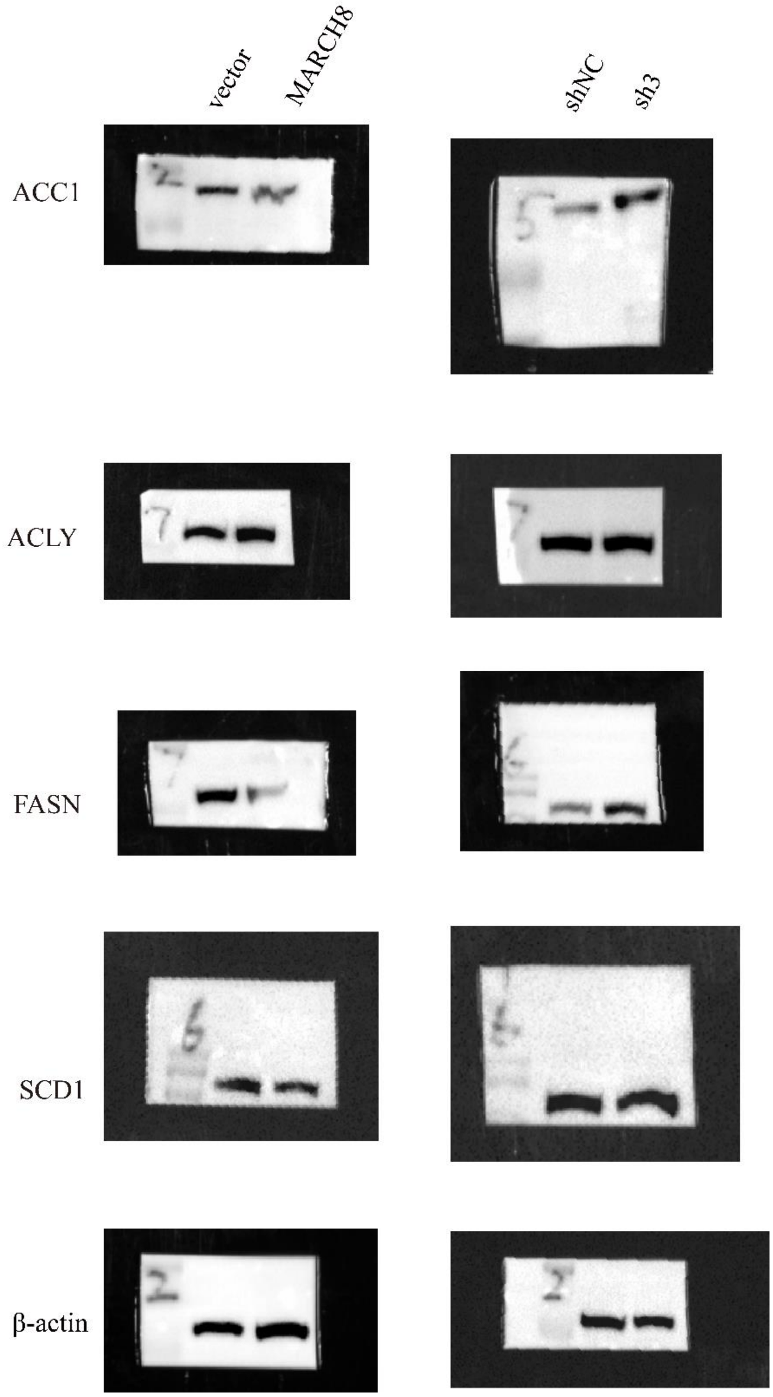

# Fig 5

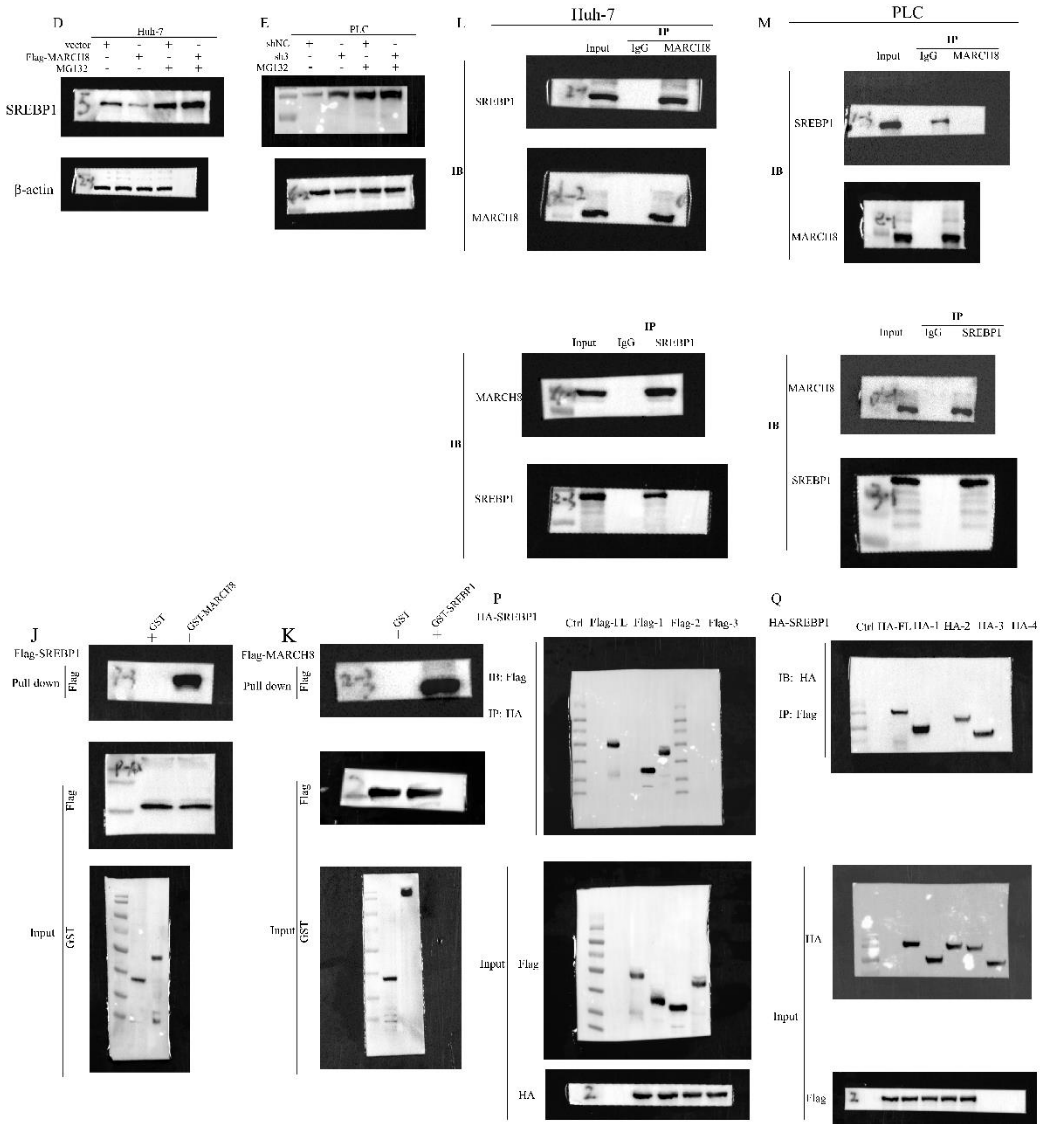
D
Huh-7
vector
Flag-MARCH8
MG132
SREBP1
β-actin
E
PLC
shNC
sh3
MG132
L
Huh-7
Input
IgG
MARCH8
IP
IB
SREBP1
MARCH8
M
PLC
Input
IgG
MARCH8
IP
IB
SREBP1
MARCH8
Input
IgG
SREBP1
IP
IB
MARCH8
SREBP1
J
Flag-SREBP1
GST
GST-MARCH8
Pull down
Flag
Input
GST
K
Flag-MARCH8
GST
GST-SREBP1
Pull down
Flag
Input
GST
P
HA-SREBP1
Ctrl
Flag-FL
Flag-1
Flag-2
Flag-3
IB: Flag
IP: HA
Input
Flag
HA
Q
HA-SREBP1
Ctrl
HA-FL
HA-1
HA-2
HA-3
HA-4
IB: HA
IP: Flag
Input
HA
Flag

**Fig 6**

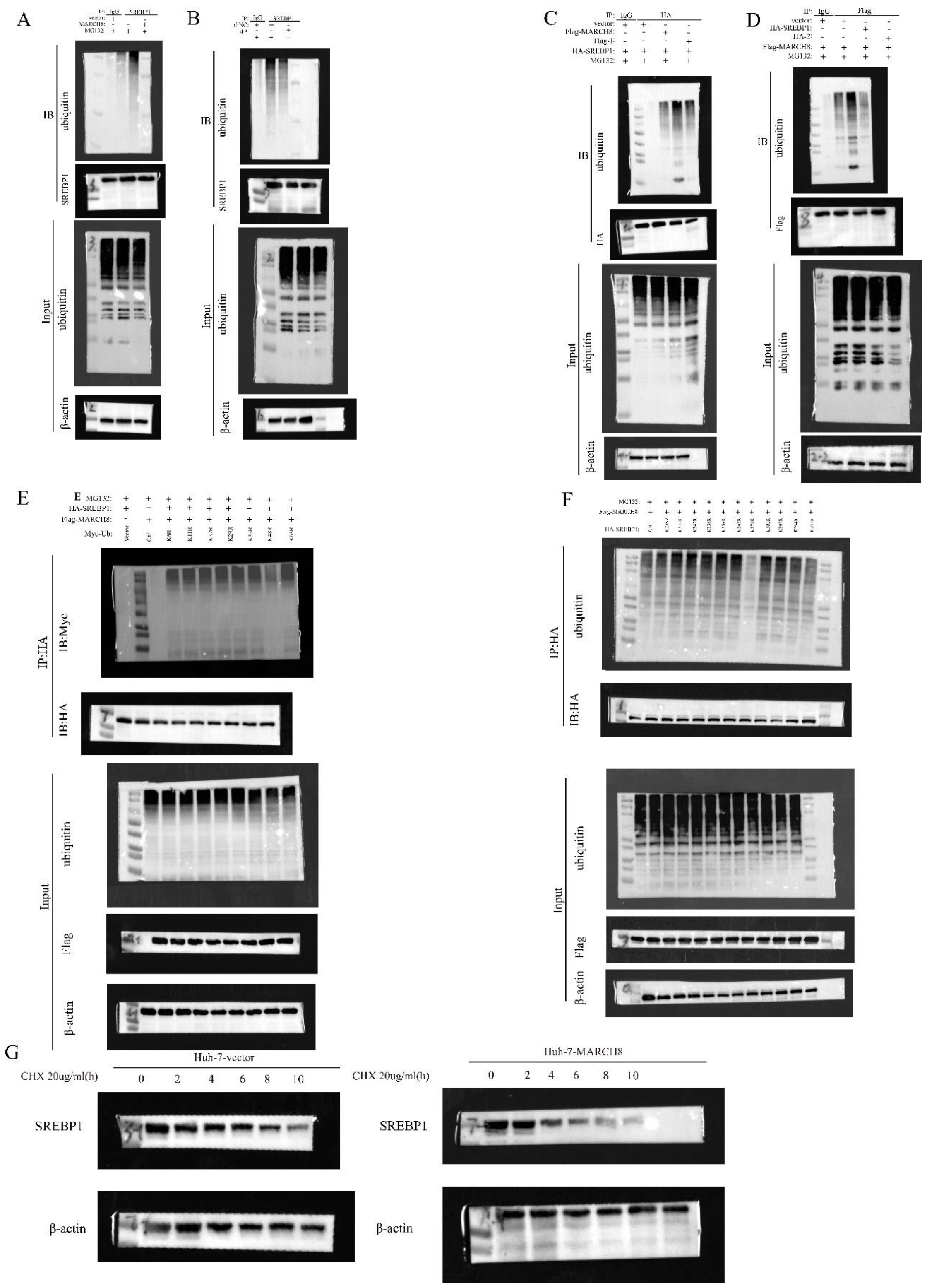
A
B
C
D
E
F
G
IB
ubiquitin
SREBP1
Input
β-actin
HA
Flag
IP:HA
IB:Myc
IB:HA
Huh-7-vector
Huh-7-MARCH8
CHX 20ug/ml(h)
0
2
4
6
8
10

**Fig 7**

A

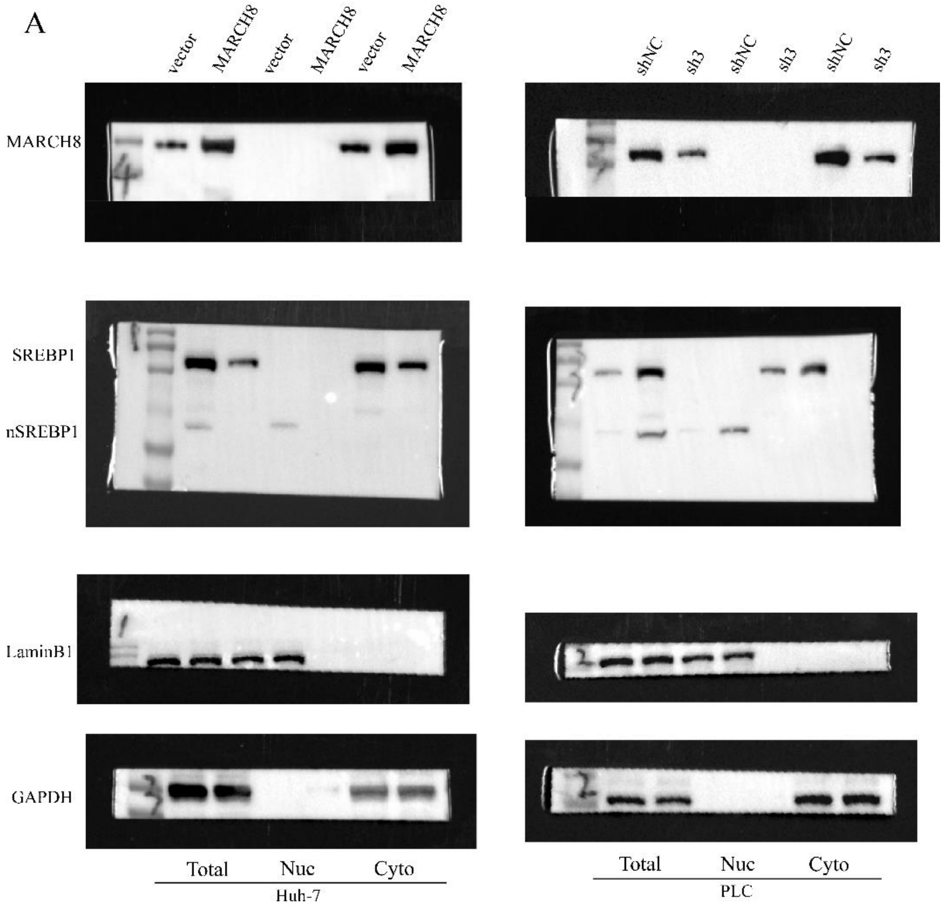
vector
MARCH8
vector
MARCH8
vector
MARCH8
shNC
sh3
shNC
sh3
shNC
sh3
MARCH8
SREBP1
nSREBP1
LaminB1
GAPDH
Total
Nuc
Cyto
Huh-7
Total
Nuc
Cyto
PLC

J

K

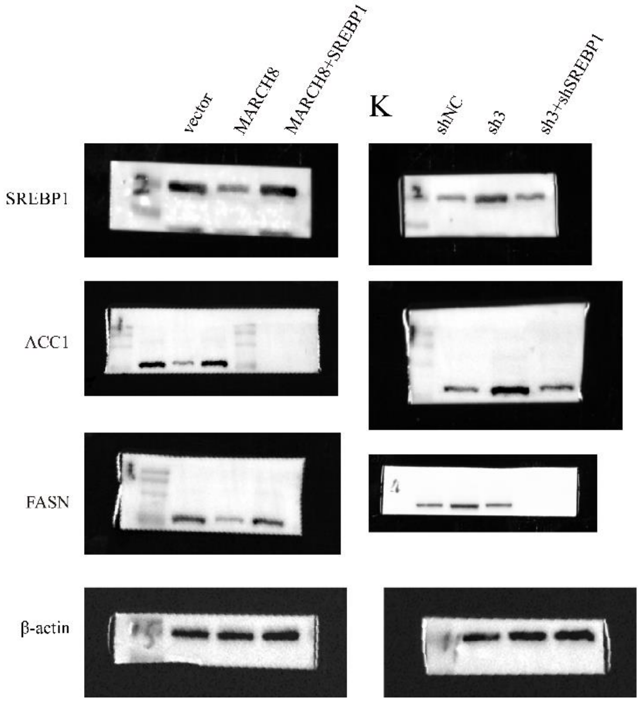
vector
MARCH8
MARCH8+SREBP1
shNC
sh3
sh3+shSREBP1
SREBP1
ACC1
FASN
β-actin

**Supp Fig 1**

A

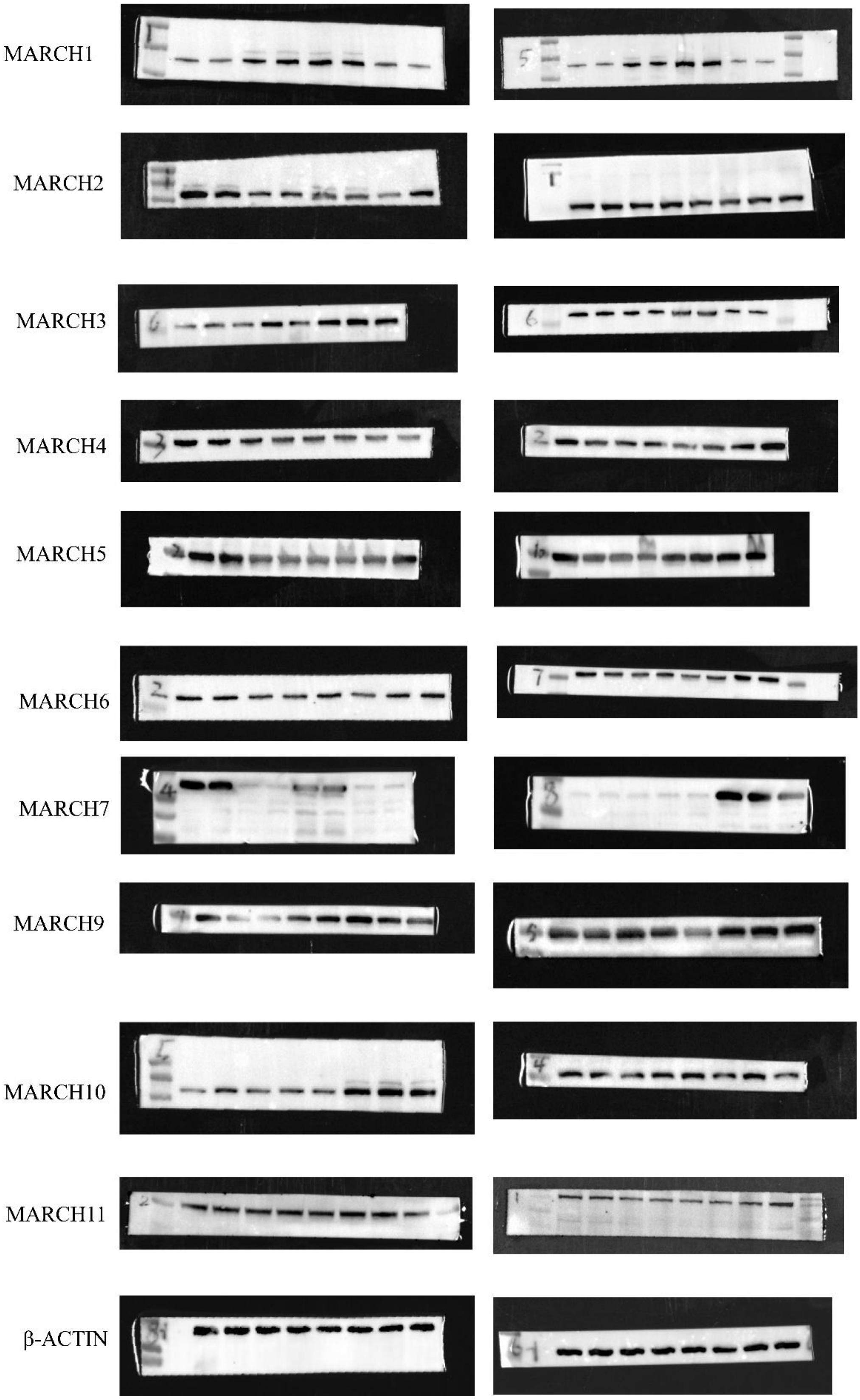
MARCH1
MARCH2
MARCH3
MARCH4
MARCH5
MARCH6
MARCH7
MARCH9
MARCH10
MARCH11
β-ACTIN

## Supp Fig 2

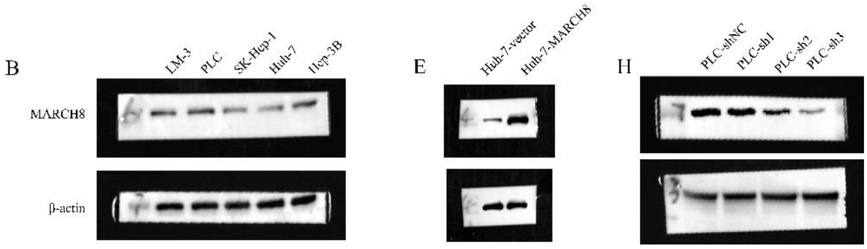


## Supp Fig 3

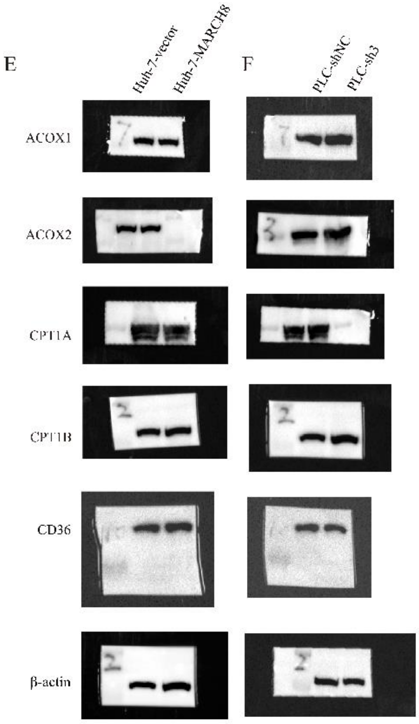


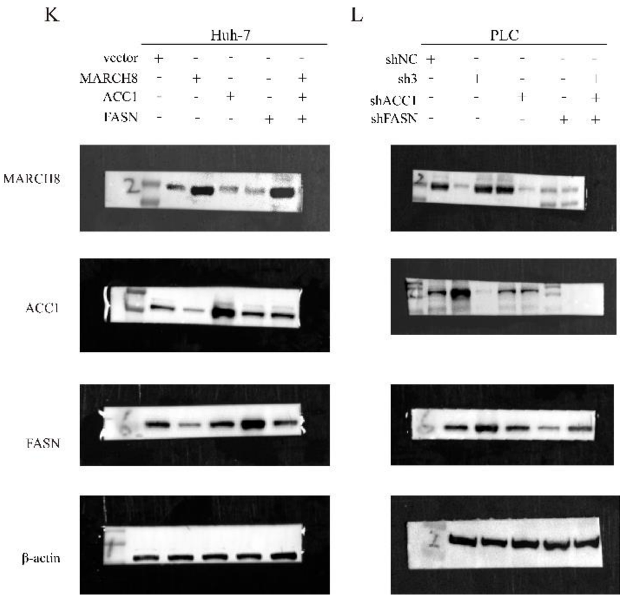

# Supp Fig 4

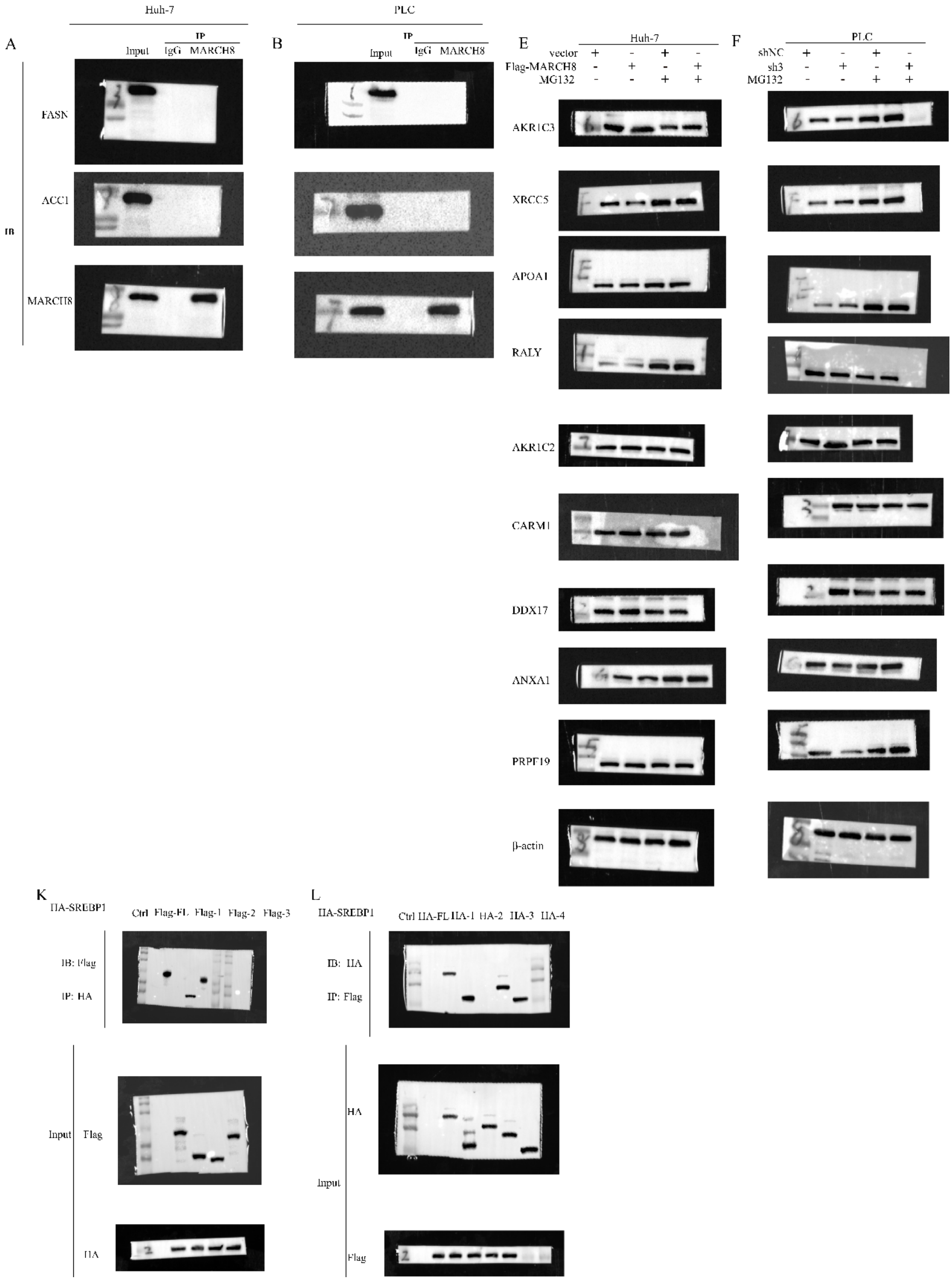
Huh-7
PLC
A
B
IP
Input
IgG
MARCH8
FASN
ACC1
MARCH8
IB
E
Huh-7
vector + - + -
Flag-MARCH8 - + - +
MG132 - - + +
F
PLC
shNC + - + -
sh3 - + - +
MG132 - - + +
AKR1C3
XRCC5
APOA1
RALY
AKR1C2
CARM1
DDX17
ANXA1
PRPF19
β-actin
K
HA-SREBP1
Ctrl
Flag-FL
Flag-1
Flag-2
Flag-3
IB: Flag
IP: HA
Input
Flag
HA
L
HA-SREBP1
Ctrl
HA-FL
HA-1
HA-2
HA-3
HA-4
IB: HA
IP: Flag
HA
Input
Flag

## Supp Fig 5

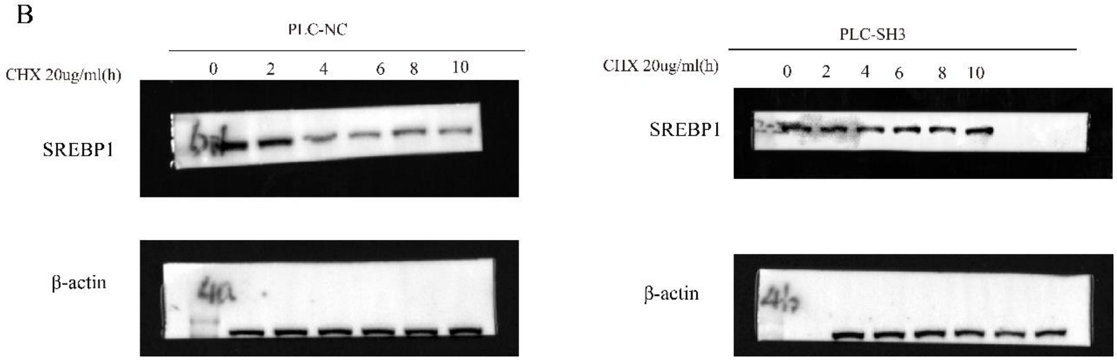


## Supp Fig 6

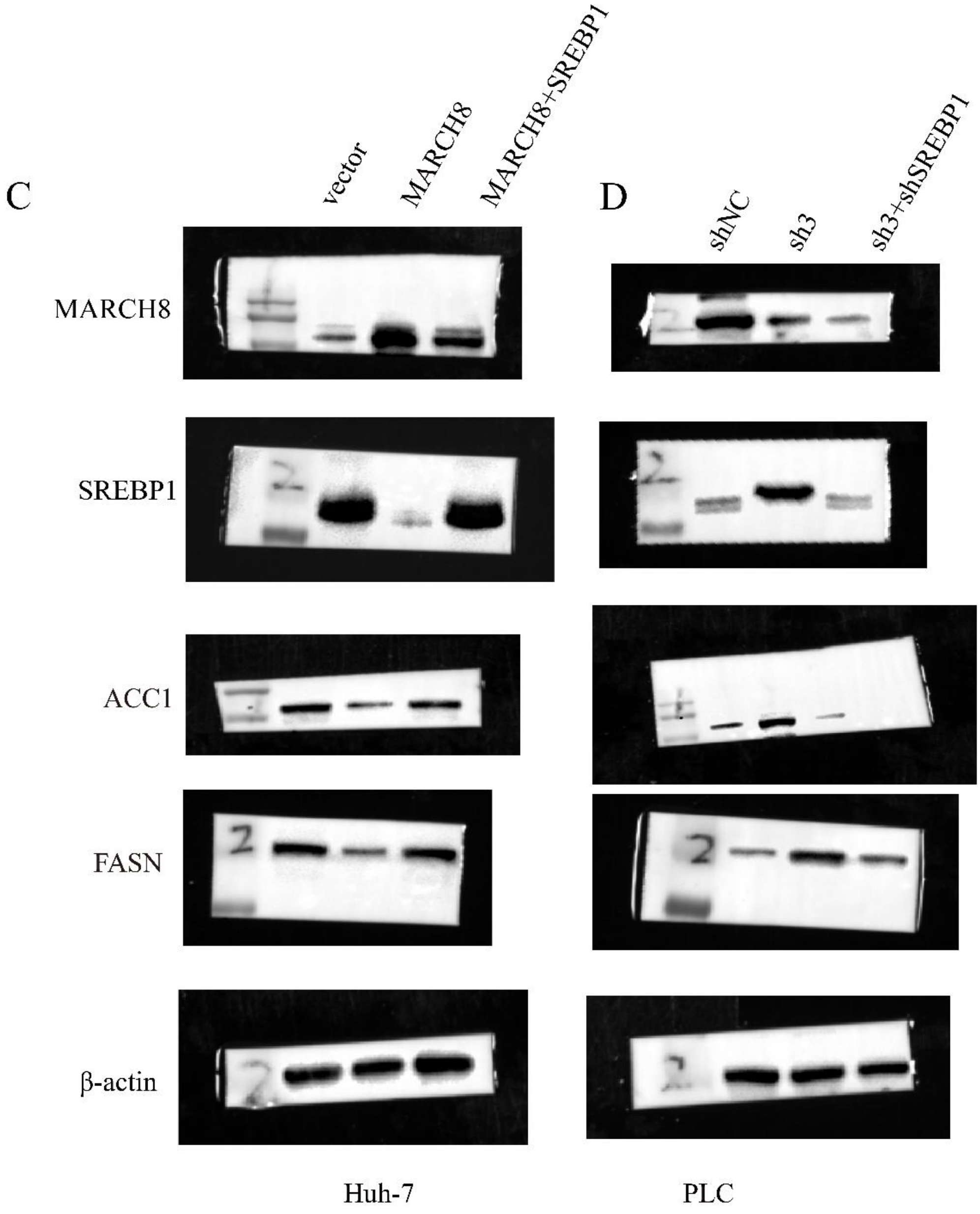

Supplement: Supplementary file 3 — Original Western blot [file 41419_2025_7707_MOESM3_ESM.pdf]
